# Structural insights into choline-*O*-sulfatase reveal the molecular determinants for ligand binding

**DOI:** 10.1107/S2059798322003709

**Published:** 2022-04-26

**Authors:** Jose Antonio Gavira, Ana Cámara-Artigas, Jose Luis Neira, Jesús M. Torres de Pinedo, Pilar Sánchez, Esperanza Ortega, Sergio Martinez-Rodríguez

**Affiliations:** a Laboratorio de Estudios Cristalográficos, CSIC, Armilla, 18100 Granada, Spain; b Department of Chemistry and Physics, University of Almería, Agrifood Campus of International Excellence (ceiA3), Research Centre for Agricultural and Food Biotechnology (BITAL), Carretera de Sacramento s/n, Almería, 04120, Spain; c IDIBE, Universidad Miguel Hernández, 03202 Elche (Alicante), Spain; d Instituto de Biocomputación y Física de Sistemas Complejos, Joint Units IQFR–CSIC–BIFI and GBsC–CSIC–BIFI, Universidad de Zaragoza, 50009 Zaragoza, Spain; e Departamento de Bioquímica y Biología Molecular III e Inmunología, Universidad de Granada, 18071 Granada, Spain

**Keywords:** choline, sulfatases, conformational gating, alkaline phosphatases, promiscuity

## Abstract

Choline-*O*-sulfatase (COSe; EC 3.1.6.6) is a member of the alkaline phosphatase (AP) superfamily, and its natural function is to hydrolyze choline-*O*-sulfate into choline and sulfate. Despite its natural function, the major interest in this enzyme resides in the landmark catalytic/substrate promiscuity of sulfatases, which has led to attention in the biotechnological field due to their potential in protein engineering. In this work, an in-depth structural analysis of wild-type *Sinorhizobium* (*Ensifer*) *meliloti* COSe (SmeCOSe) and its C54S active-site mutant is reported. The binding mode of this AP superfamily member to both products of the reaction (sulfate and choline) and to a substrate-like compound are shown for the first time. The structures further confirm the importance of the C-terminal extension of the enzyme in becoming part of the active site and participating in enzyme activity through dynamic intra-subunit and inter-subunit hydrogen bonds (Asn146^*A*^–Asp500^*B*^–Asn498^*B*^). These residues act as the ‘gatekeeper’ responsible for the open/closed conformations of the enzyme, in addition to assisting in ligand binding through the rearrangement of Leu499 (with a movement of approximately 5 Å). Trp129 and His145 clamp the quaternary ammonium moiety of choline and also connect the catalytic cleft to the C-terminus of an adjacent protomer. The structural information reported here contrasts with the proposed role of conformational dynamics in promoting the enzymatic catalytic proficiency of an enzyme.

## Abstract 2

The first structures of a choline-*O*-sulfatase bound to different ligands are reported.

## 1. Introduction

**Figure 1:**
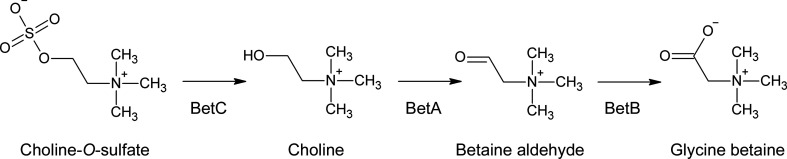
Three-step biosynthetic pathway of glycine betaine starting from choline-*O*-sulfate. BetC, choline-*O*-sulfatase; BetB, betaine aldehyde dehydrogenase; BetA, choline dehydrogenase.

More than half a century ago, the sulfoester choline-*O*-sulfate (COS) attracted the attention of the scientific community, prompted by the high levels at which this compound accumulates in some organisms (more than 0.2% of the total dry weight in *Aspergillus*, *Penicillium*, *Rocella* and *Gelida* species; Harada & Spencer, 1960 ▸), initially suggesting a putative role in sulfur storage (Nissen & Benson, 1961 ▸; Hussey *et al.*, 1965 ▸; Spencer *et al.*, 1968 ▸). During recent decades, various studies have investigated two different alternative roles of this quaternary ammonium compound: (i) as a reservoir not only for sulfur, but also for carbon and nitrogen, preventing cell starvation during elemental depletion events, or (ii) as an osmoprotectant (Cregut *et al.*, 2014 ▸; Galvão *et al.*, 2006 ▸; Osterås *et al.*, 1998 ▸). COS can be oxidized to glycine-betaine by various microorganisms using a three-step enzymatic cascade codified in the *betICBA* operon (Fig. 1 ▸). Three enzymes are responsible for this metabolic pathway: choline-*O*-sulfatase (BetC), betaine aldehyde dehydrogenase (BetB) and choline dehydrogenase (BetA) (Osterås *et al.*, 1998 ▸). An additional regulatory protein (BetI) regulates the expression of *bet* genes in response to choline availability (Mandon *et al.*, 2003 ▸). Choline-*O*-sulfatase (COSe; EC 3.1.6.6) is responsible for the first step of this pathway, consisting of the breakdown of COS into choline. This enzyme belongs to the S1 family of sulfatases (subfamily 12) according to the SulfAtlas database (Barbeyron *et al.*, 2016 ▸).

The presence of COSe in *Pseudomonas* (Lucas *et al.*, 1972 ▸; Jovcic *et al.*, 2011 ▸), *Aspergillus* (Scott & Spencer, 1968 ▸) and *Penicillium* (Lucas *et al.*, 1972 ▸) species was reported several decades ago. More recently, studies of the existence of COSe enzymes in various microorganisms have been reported (Cregut *et al.*, 2014 ▸; Lidbury *et al.*, 2015 ▸). The recombinant enzyme from *Sinorhizobium meliloti* (SmeCOSe) has been biochemically studied in some detail (Sánchez-Romero & Olguin, 2015 ▸), followed by a profound study of its substrate promiscuity, together with the first COSe X-ray structure (PDB entry 6fny; 2.8 Å resolution; van Loo *et al.*, 2018 ▸). Despite this seminal work, the modest resolution of this structure precluded precise determination of the molecular determinants governing the enzymatic activity of SmeCOSe. This enzyme belongs to the alkaline phosphatase (AP) superfamily, which has come to the attention of the scientific community during the last decade due to the landmark catalytic promiscuity of sulfatases and phosphatases (van Loo, Bayer *et al.*, 2019 ▸; Pabis & Kamerlin, 2016 ▸; Luo *et al.*, 2012 ▸; van Loo, Berry *et al.*, 2019 ▸; Pabis *et al.*, 2016 ▸; Miton *et al.*, 2018 ▸; Duarte *et al.*, 2013 ▸; Barrozo *et al.*, 2015 ▸; Mohamed & Hollfelder, 2013 ▸).

In this work, we provide an in-depth structural analysis of SmeCOSe together with the first ligand-bound structures of this enzyme, providing clues to the molecular basis of its catalytic promiscuity. Our different ligand-bound structures confirm key roles of FGly54 (formyl-glycine), Asn75, Trp129, His145, His201 and Lys309 in ligand positioning, together with Asp386, which might also assist during product release. Contrary to the previous hypothesis, we confirm the general ligand-binding strategy observed for other choline-binding proteins, in which Trp129 and His145 belonging to a conserved β-hairpin are responsible for ligand accommodation. We further demonstrate the dynamic character of various residues in the catalytic environment as a result of ligand binding, including dynamic intra-subunit and inter-subunit hydrogen bonds (Asn146^*A*^–Asp500^*B*^–Asn498^*B*^) that are responsible for the open/closed conformations of the enzyme, while assisting in ligand binding through the rearrangement of Leu499, with a displacement of approximately 5 Å. Finally, based on information derived from our structures, we argue against the proposed role of conformational dynamics in promoting the enzymatic catalytic proficiency of SmeCOSe.

## 2. Materials and methods

### 2.1. Materials

All chemicals were of analytical grade and were used without further purification. Ni–NTA resin was purchased from Qiagen, *p*-nitrophenyl sulfate (pNPS) from Sigma and choline chloride from Alfa Aesar. Choline-*O*-sulfate was prepared as reported previously (Stevens & Vohra, 1955 ▸), with slight modifications. Briefly, 4 g choline chloride was added to 10 ml 98% sulfuric acid in a round flask and the mixture was heated under reflux for 4 h. The solution was cooled at room temperature (RT), transferred into a glass crystallizer and mixed with 80 ml absolute ethanol. The mixture was stored overnight at 4°C. Recrystallization was conducted in 75% ethanol.

### 2.2. Microbes and culture conditions

*Sinorhizobium* (*Ensifer*) *meliloti* CECT4857 was used as a possible donor of the choline sulfatase gene (*betC*). It was grown at 30°C for 24 h on nutrient broth/agar I plates (1% peptone, 0.5% beef extract, 0.5% NaCl pH 7.2, 1.5% agar). *Escherichia coli* DH5α cells were used for cloning and *E. coli* BL21 (DE3) cells were used for overexpression.

### 2.3. Cloning and sequence analysis of SmeCOSe

Colony PCR was carried out to amplify the choline sulfatase (*betC*) gene from *S. meliloti*. The amplified DNA and pET-21b(+) plasmid (Novagen) were digested using NdeI and HindIII (Fermentas) and purified from agarose gel using an E.Z.N.A. Gel Extraction Kit (Omega Bio-tek). The DNA fragments were ligated to create plasmid pSERQF5. The resulting construct allows the production of recombinant SmeCOSe fused to a His_6_ tag at the C-terminus. Sequencing confirmed 99.9% identity to the choline sulfatase gene from *S. meliloti* GR4 (GenBank accession No. CP003933.2), showing a unique nucleotide change resulting in an F105L mutation in the amino-acid sequence. The sequence corresponding to choline sulfatase from *S. meliloti* CECT 4857 was deposited in GenBank (accession No. MH208481).

### 2.4. Mutagenesis of Cys54 of SmeCOSe

Mutagenesis was performed with a QuikChange II site-directed mutagenesis kit from Stratagene following the manufacturer’s protocol, using the pSERQF5 plasmid as the template. Mutation was confirmed by sequencing. The plasmids containing SmeCOSe with a C54A or C54S mutation were transformed into *E. coli* BL21 (DE3) cells and protein overexpression and purification were carried out as described for the wild-type (WT) enzyme.

### 2.5. Overexpression and purification of WT and mutated SmeCOSe species

A single colony was transferred into 10 ml LB medium supplemented with 100 µg ml^−1^ ampicillin in a 50 ml flask and the culture was incubated overnight at 37°C with shaking. 1 l LB supplemented with 100 µg ml^−1^ ampicillin was inoculated with 10 ml of the overnight culture in a 2 l flask. After 2–3 h of incubation at 37°C with vigorous shaking, when the OD_600_ of the medium reached a value of 0.4–0.6, isopropyl β-d-1-thiogalactopyranoside (IPTG) was added to a final concentration of 0.2 m*M* and the culture was continued at 32°C overnight. The cells were collected by centrifugation (5000 rev min^−1^, 20 min, 4°C) and stored at −20°C until use.

The pellet was resuspended in wash buffer (WB; 50 m*M* sodium phosphate pH 8.0, 300 m*M* sodium chloride) and the cell walls were disrupted on ice by pulsed sonication using a Branson Digital Sonifier (six periods of 30 s, pulse mode 0.5 and sonic power 60%). The resulting lysate was subjected to centrifugation (13 000 rev min^−1^, 30 min, 4°C) in order to obtain the corresponding soluble fraction. The pellet was discarded and the supernatant was applied onto a column packed with Ni–NTA resin (2 ml bed volume, Qiagen) previously equilibrated with WB. The column was washed with 20 ml WB and the protein was eluted with 5 ml elution buffer (EB; 50 m*M* sodium phosphate pH 8.0, 300 m*M* sodium chloride, 250 m*M* imidazole). The nickel column eluate was directly loaded onto a Superdex 200 16/60 gel-filtration column in an ÄKTAprime FPLC system (GE Healthcare) using 20 m*M* Tris–HCl pH 8.0 as the running buffer. The fractions corresponding to the sulfatase were pooled, concentrated to 15–20 mg ml^−1^ using a Centricon centrifugation system with a 30 kDa cutoff membrane and dialyzed against 20 m*M* Tris–HCl pH 8.0 at 4°C. SmeCOSe samples were stored at −80°C until use. Protein purity was verified by SDS–PAGE. Protein concentration was determined spectrophotometrically at 280 nm using a theoretical extinction coefficient of 97 750 *M* ^−1^ cm^−1^.

### 2.6. Enzymatic assays

Activity with choline sulfate was tested by the Reineckate method (Engel, 1942 ▸). However, due to its ease of utilization, pNPS was used as the general substrate for enzyme characterization. A 70 m*M* stock solution in 20 m*M* Tris pH 8.0 was used. Changes in the absorbance at 405 nm as a result of *p*-nitrophenol (pNP) formation were followed spectrophoto­metrically in a Nanodrop instrument (ThermoFisher).

### 2.7. Determination of the oligomerization state of SmeCOSe

Size-exclusion chromatography (SEC) and dynamic light scattering (DLS) were used to test the oligomerization state of recombinant WT SmeCOSe. SEC experiments were carried out with 100 µl samples of SmeCOSe (0.1–1 mg ml^−1^) loaded onto a Tricorn Superdex 200 gel-filtration column in an ÄKTAprime FPLC system (GE Healthcare) using 20 m*M* Tris–HCl pH 8.0 as the running buffer.

DLS experiments were performed with a Zetasizer Nano ZS (Malvern Instruments, Worcestershire, United Kingdom; 4 mV He–Ne laser, λ_0_ = 633 nm and Θ = 173°) using a thermostatized 12 µl quartz sample cuvette at 20°C. Protein samples were prepared in 20 m*M* Tris–HCl pH 8.0. The sample concentration was 20 µ*M* (in protomer units). All solutions were filtered immediately before measurements; protein samples were centrifuged for 30 min at 14 000 rev min^−1^ to remove any aggregates and dust. Data were analyzed using the manufacturer’s software: the hydrodynamic radius, *R*_h_, and molecular mass, *M*, were determined from the Stokes–Einstein equation, assuming a spherical shape for the protein.

### 2.8. Thermal shift assays

Thermal shift assays (TSAs) were carried out using a QuantStudio 3 qPCR (Applied Biosystems, ThermoFisher). Thermal denaturation measurements were monitored by measuring the changes in fluorescence as a result of SYPRO binding. Thermal unfolding curves were collected from 25 to 95°C at a scan rate of 3°C min^−1^. Three replicates were conducted in all cases, with a final concentration of 8× SYPRO. Thermal denaturation of WT SmeCOSe (1.0 mg ml^−1^) was assayed in various buffers at 10 m*M* (pH 4.0–10). Ligand binding was assayed by incubation of WT or C54S SmeCOSe (0.5–1.0 mg ml^−1^ in 20 m*M* Tris pH 8.0) with choline, sulfate and COS (0.02–200 m*M*) or pNP (0.1–5 m*M*). After 10 min of incubation at RT, denaturation experiments were conducted as described above. Thermal denaturation midpoints ( 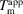 ) were calculated using the *Protein Thermal Shift* software 1.3 (Applied Biosystems, ThermoFisher), adjusting the data to the Boltzmann equation. Since the temperature melts were irreversible, the 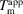 values should only be considered to be a qualitative measure of the similar stability of the WT and mutated species. Other sulfate-containing (HEPES, CAPS, CAPSO, CHES and MOPS) or cyclic (pyperazidine, imidazole and pyridine) compounds were tested for ligand binding to the C54S mutant.

### 2.9. Far-UV CD spectroscopy

The secondary structures of the WT and C54A/C54S SmeCOSe species were compared using far-UV circular-dichroism (CD) spectra recorded with a Jasco J850 CD spectrometer equipped with a Jasco PTC-423S/15 Peltier accessory. Protein concentrations were 2–6 µ*M* in various buffers at 10 m*M* (see Section 2.6). Experiments were acquired with a response time of 8 s, a bandwidth of 1 nm and a step resolution of 0.2 nm. CD measurements were taken at 25°C using a 1 mm path-length cuvette. Spectra were acquired from 250 to 190 nm at a scan rate of 50 nm min^−1^ and were averaged over five scans. Thermal experiments were performed at constant heating rates of 60°C h^−1^ (1°C min^−1^) with a response time of 8 s. Thermal scans were collected by following the changes in ellipticity at 222 nm, typically from 25 to 70°C. Data were collected every 0.2°C, with a response time of 8 s and a bandwidth of 1 nm.

### 2.10. Crystallization

Recombinant C-terminally His_6_-tagged WT SmeCOSe (18–20 mg ml^−1^ in 20 m*M* Tris–HCl pH 8.0) was used to perform initial crystallization screening with Crystal Screen and Crystal Screen 2 from Hampton Research. Initial crystals were obtained by the vapor-diffusion technique using a sitting-drop setup at 20°C, with drops made by mixing equal volumes (1 µl) of protein solution and reservoir solution. Optimization was attempted by slightly varying the conditions of precipitant concentration and pH around the original hits. Co-crystallization of WT and C54S SmeCOSe (18–20 mg ml^−1^ in 20 m*M* Tris–HCl pH 8.0) with choline (10 m*M*) or HEPES (100 m*M*) was also assayed following the same strategy.

### 2.11. Data collection and refinement

Target crystals (Supplementary Fig. S1) were fished out of the drop with a loop and transferred into a 5 µl drop of mother solution containing 15–20%(*v*/*v*) glycerol. After soaking for less than 60 s, the crystals were flash-cooled in liquid nitrogen and stored until data collection. X-ray diffraction data were collected on the ID23-1, ID30A1 and ID30B beamlines at the European Synchrotron Radiation Facility (ESRF), Grenoble, France. Diffraction frames were indexed and integrated using *XDS* (Kabsch, 2010 ▸) and were scaled, reduced and merged with *AIMLESS* (Evans & Murshudov, 2013 ▸) from the *CCP*4 suite (Winn *et al.*, 2011 ▸). The crystal structure of SmeCOSe was determined by the molecular-replacement method with *MOLREP* (Vagin & Teplyakov, 2010 ▸) using the putative arylsulfatase from *E. coli* as the search model (PDB entry 3ed4; New York SGX Research Center for Structural Genomics, unpublished work). Refinement was carried out with *Phenix* (Adams *et al.*, 2010 ▸) and *REFMAC* (Murshudov *et al.*, 2011 ▸) with cycles of manual rebuilding using *Coot* (Emsley *et al.*, 2010 ▸) and was finalized by including several cycles of refinement applying TLS parameterization (Painter & Merritt, 2006 ▸). Model quality was followed with *MolProbity* (Chen *et al.*, 2010 ▸) within the *Phenix* package (Liebschner *et al.*, 2019 ▸). Data-collection and refinement statistics are summarized in Table 1 ▸.

### 2.12. Sequence and structure analysis

*Clustal Omega* (Madeira *et al.*, 2019 ▸) and *BioEdit* (Hall, 1999 ▸) were used for multiple sequence alignment and phylogenetic analysis. The *DALI* server (Holm, 2020 ▸) was used to search for other members of the AP superfamily with a similar fold to that of the SmeCOSe structure. The *PISA* server was used to calculate probable assemblies of SmeCOSe (Krissinel & Henrick, 2007 ▸). Molecular-dynamics simulations were carried out with the *CABS-flex* 2.0 server (Kuriata *et al.*, 2018 ▸). Graphical representations of 3D structural models were obtained with *PyMOL* (DeLano, 2002 ▸).

## 3. Results and discussion

### 3.1. Characterization of SmeCOSe

**Figure 2:**
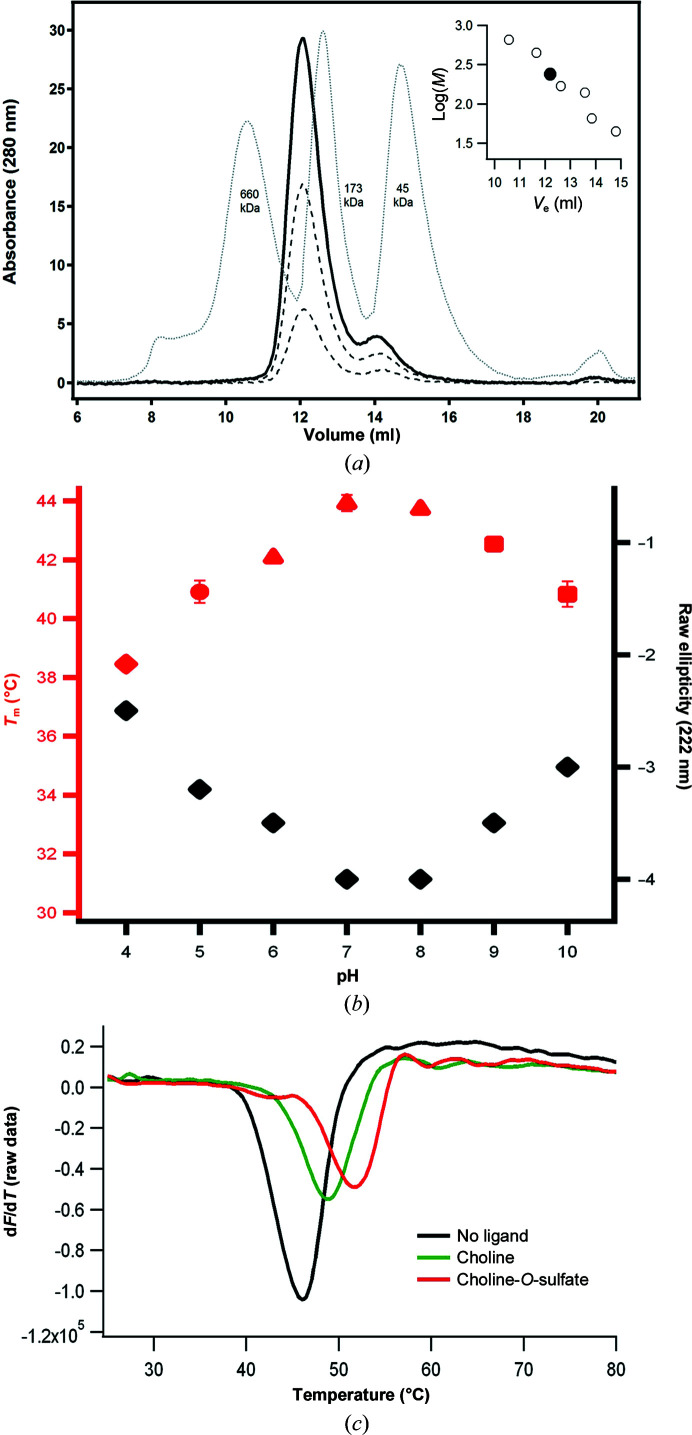
Characterization of SmeCOSe samples. (*a*) SEC experiments carried out with SmeCOSe in 20 m*M* Tris–HCl pH 8.0 at three concentrations (solid black line and dashed lines). Thyroglobulin (660 kDa), glucose isomerase (173 kDa) and ovoalbumin (45 kDa) standards are also shown. The inset represents the fitting used to estimate the oligomerization of SmeCOSe (black point; estimated molecular mass of 201 ± 6 kDa). (*b*) Effect of pH on the apparent thermal denaturation midpoint of SmeCOSe (red symbols, left axis) and the raw ellipticity at 222 nm, which is indicative of α-helical secondary structure (far UV-CD, black diamonds, right axis). (*c*) TSA experiments showing the increase in the 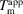 of the C54S SmeCOSe mutant in the presence of choline-*O*-sulfate and choline.

Since differences in oligomeric state, post-translational modification (PTM) and enzymatic activity have previously been reported (van Loo *et al.*, 2018 ▸, Sánchez-Romero & Olguin, 2015 ▸), we decided to further characterize our protein preparations for comparison with previous results. SmeCOSe samples were more than 95% pure, as judged from SDS–PAGE (Supplementary Fig. S2*a* ). SEC experiments yielded a major peak with an estimated molecular mass of 201 ± 6 kDa (Fig. 2 ▸ *a*), suggesting a tetrameric arrangement. DLS experiments performed at pH 8.0 show a single peak in the particle-size distribution by intensity, with a hydrodynamic radius of 5.7 ± 2.1 nm (Supplementary Fig. S2*b* ), which corresponds to a globular protein size of 200 kDa, also supporting a tetrameric quaternary arrangement. The *PISA* server further supports a tetrameric quaternary structure (Krissinel & Henrick, 2007 ▸). These results are in agreement with previously reported oligomeric characterization of SmeCOSe (van Loo *et al.*, 2018 ▸). Far UV-CD spectra showed changes in the raw ellipticity at 222 nm (which is usually assumed to mirror the percentage of α-helical secondary structure) of WT SmeCOSe below pH 7.0 and above pH 9.0 (Fig. 2 ▸ *b*) due to acidic and basic denaturation, respectively. The TSAs were concentration dependent, as is usual for irreversible denaturation processes. The highest apparent thermal midpoints ( 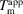 ) were obtained at pH 7.0–8.0 (Fig. 2 ▸ *b*), in agreement with the kinetic profiles obtained previously for SmeCOSe (van Loo *et al.*, 2018 ▸; Sánchez-Romero & Olguin, 2015 ▸). These findings suggest that the decrease in the kinetic parameters below pH 7.0 is most likely to arise from changes in the secondary structure of the enzyme. TSAs showed an increase in the 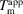 of WT SmeCOSe in the presence of the products of the reaction (increases of 0.8°C for sulfate and 1.3°C for choline); these differences were larger for the C54S mutant (Fig. 2 ▸ *c*).

SmeCOSe has been shown to efficiently hydrolyze pNPS (van Loo *et al.*, 2018 ▸; Sánchez-Romero & Olguin, 2015 ▸; Supplementary Fig. S3) in addition to its natural substrate (COS). A *k*_cat_ of 0.22 ± 0.03 s^−1^ and a *K*_m_ of 45.7 ± 12.5 m*M* were obtained for pNPS at pH 7.0, similar to the results reported previously (a *k*_cat_ of 0.22 ± 0.01 s^−1^ and a *K*_m_ of 19.0 ± 2.0 m*M* at pH 6.0; van Loo *et al.*, 2018 ▸). No detectable activity was observed for the C54A mutant, whereas the C54S mutant maintained a residual activity below 1%. No significant differences were found in the far-UV CD spectra of the mutants or in the thermal midpoints determined by de­naturation experiments (Supplementary Fig. S4); these results suggest that their secondary structures are highly similar to that of the WT. TSAs also showed an increase in the 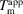 of C54S SmeCOSe in the presence of COS and choline, and also with different sulfate-containing ligands (Fig. 2 ▸ *c* and Supplementary Fig. S5). Thus, our results confirm the properties shown by the SmeCOSe preparations of Hollfelder and coworkers (van Loo *et al.*, 2018 ▸).

### 3.2. Overall analysis of SmeCOSe structures

Shortly before we deposited our initial WT SmeCOSe 3D models (PDB entries 6g5z and 6g60), Hollfelder and coworkers published a seminal work on the substrate promiscuity and putative evolution of choline sulfatases within the AP superfamily (van Loo *et al.*, 2018 ▸), including the only available structure of a COSe reported to date (PDB entry 6fny, 2.8 Å resolution). The COSe from *S. meliloti* CECT4857 used in this work presents a single L105F mutation with respect to that protein (Genbank Accession No. QAT12853). WT SmeCOSe crystals alone or co-crystallized with choline were grown in 1.0 *M* Li_2_SO_4_, 0.1 *M* Tris pH 7.0, whereas the HEPES-bound C54S SmeCOSe structure was obtained from crystals obtained using 1.5 *M* Li_2_SO_4_, 0.1 *M* HEPES pH 7.5; they belonged to space group *C*121 (Table 1 ▸). On the other hand, C54S SmeCOSe was co-crystallized with choline using 0.2 *M* sodium acetate trihydrate, 0.1 *M* Tris pH 8.5, 30%(*w*/*v*) polyethylene glycol 4000, yielding crystals belonging to space group *P*12_1_1 (Table 1 ▸). The crystals used for data collection (Supplementary Fig. S1) diffracted far beyond those characterized previously (for example, PDB entry 6g5z at 1.84 Å resolution), allowing a more reliable fitting of the SmeCOSe structures and the deciphering of structural and mechanistic features that had not been correctly assigned before. We also obtained structures of the C54S active-site mutant bound to two different ligands (PDB entries 7pth and 7ptj), also achieving a resolution of 1.8 Å.

Comparison of chains *A* among our structures and PDB entry 6fny (residues Lys5–His512, comprising 2032 backbone atoms) yielded r.m.s.d.s in the range 0.15–0.37 Å. The most remarkable differences were observed within segment 485–487, which is not fully defined in some of our structural models despite the higher resolution. The differences most likely arise from different crystal packing: the Lys487–Asn178 interaction in PDB entry 6fny seems to account for the better definition of this region. The SmeCOSe C-termini (Supplementary Fig. S6) seem to be an essential element for oligomerization, and the presence of a C-terminal His_6_ tag was supposed to cause the dimeric arrangement observed in previous preparations (Sánchez-Romero & Olguin, 2015 ▸). On the other hand, a tetrameric arrangement was determined in our preparations (Fig. 2 ▸ *a* and Supplementary Fig. S2*b* ) and in those characterized by van Loo *et al.* (2018 ▸). Comparison of the tetrameric arrangement of SmeCOSe in the structure solved in space group *P*12_1_1 with those in space group *C*121 showed r.m.s.d.s in the range 0.45–0.51 Å (Supplementary Fig. S7), which are a little higher than those calculated among the structures solved in space group *C*121 (0.17–0.28 Å). The His_6_ tag at the C-termini of two chains of PDB entry 7pth could be modeled, probably as a consequence of stabilization by the His519^*A*^–Glu223^*B*^ interaction, which is not observed in other structures. Small differences are observed in the overall arrangement of SmeCOSe as a result of a slight rotation of three subunits with respect to a fixed protomer, most likely arising from the different crystal packing, which is more compact for space group *P*12_1_1 (61% solvent content in *C*121 versus 49% in *P*12_1_1). On the other hand, a comparison of the two choline-bound tetrameric structures obtained in two different space groups (PDB entries 6g60 and 7pth) showed no appreciable differences in the conformation of the different loops in the overall structure (Supplementary Fig. S7), including loops in the environment of the catalytic cleft (see below).

Different members of the AP superfamily rely on a post-translationally modified cysteine found within a conserved C*X*P*X*R motif to accomplish their enzymatic task (Appel & Bertozzi, 2015 ▸; Sidhu *et al.*, 2014 ▸; Carlson *et al.*, 2008 ▸; Hanson *et al.*, 2004 ▸). Mutation of Cys54 in SmeCOSe to Ala or Ser greatly affected the enzymatic activity (see above). Whereas the presence of this PTM had already been ascertained (van Loo *et al.*, 2018 ▸), inspection of the catalytic environment of the WT SmeCOSe structure clearly shows densities shaping a hydrated formylglycine (FGly) residue (Supplementary Fig. S8*a* ). The extra densities are not present in the C54S mutant structure, further supporting this PTM (Supplementary Fig. S8*b* ). Although the modification of different recombinant sulfatases has been suggested to be brought about in *E. coli* by an unknown enzyme endogenous to the protein-production host (van Loo *et al.*, 2018 ▸), a different sulfatase-maturation system is known to occur in prokaryotes (Benjdia *et al.*, 2007 ▸).

**Figure 3:**
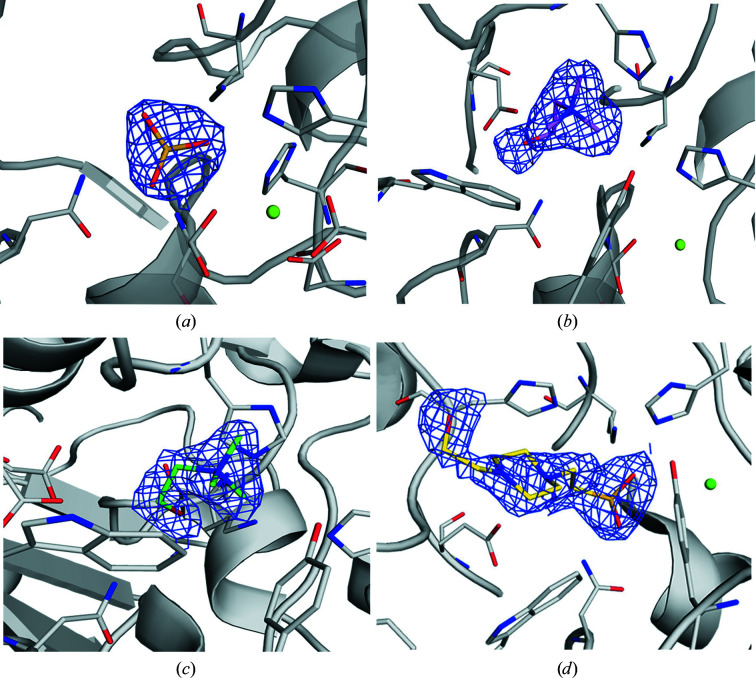
|2*F*_o_ − *F*_c_| electron-density maps contoured at 1σ of the different ligands fitted in SmeCOSe structures. (*a*) Sulfate-bound model of WT SmeCOSe (PDB entry 6g5z). (*b*) Choline-bound model of WT SmeCOSe (PDB entry 6g60). (*c*) Choline-bound model of C54S SmeCOSe (PDB entry 7pth). (*d*) HEPES-bound model of C54S SmeCOSe (PDB entry 7ptj).

Both mononuclear and binuclear metallocenters have been described in the AP superfamily (Jonas & Hollfelder, 2009 ▸). Like other arylsulfatases (Hanson *et al.*, 2004 ▸) and phosphonate monoester hydrolases (PMHs; van Loo *et al.*, 2010 ▸, van Loo, Bayer *et al.*, 2019 ▸), SmeCOse is a mononuclear metalloenzyme. As shown previously (van Loo *et al.*, 2018 ▸), Asp14, FGly54, Asp296 and His297 are at binding distances from the cation (Fig. 3 ▸). The position of the second metal in different members of this superfamily has been shown to be substituted by a highly conserved basic residue (Jonas & Hollfelder, 2009 ▸). In the case of SmeCOSe, His201 would approximately occupy this position.

### 3.3. Structural insights into ligand binding by SmeCOSe

#### 3.3.1. Sulfate binding

**Figure 4:**
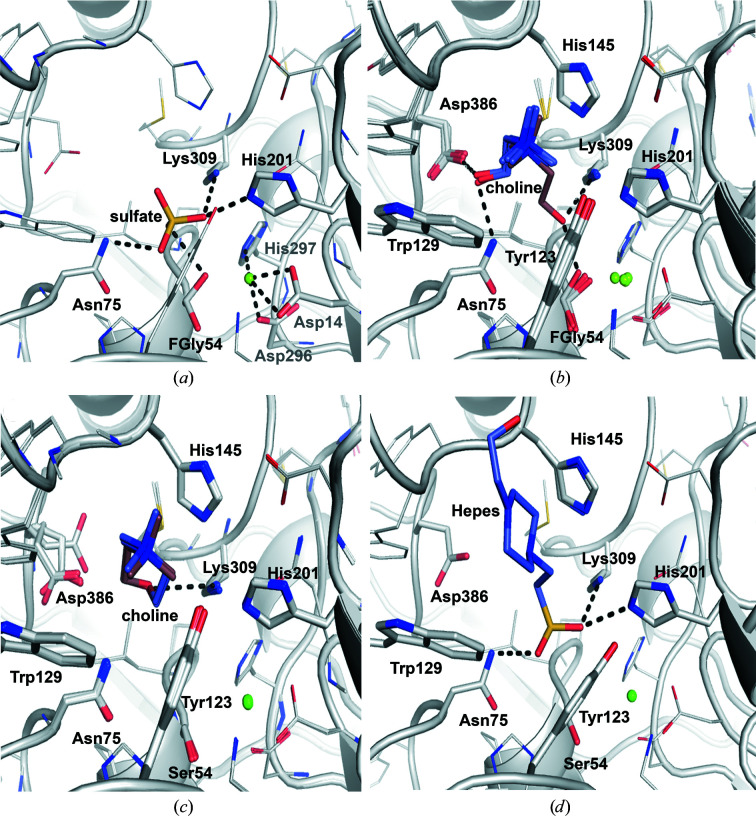
Catalytic environment of WT and C54S SmeCOSe showing different ligands. (*a*) Catalytic environment of WT SmeCOSe showing a bound sulfate molecule (PDB entry 6g5z). The catalytic cation (green) and its environment are also shown. (*b*, *c*) The choline binding environment in the different chains of WT (*b*) and C54S (*c*) SmeCOSe (PDB entries 6g60 and 7pth, respectively). Leu499 from a second protomer is omitted for clarity. Different colors for choline have been used to highlight the different orientations that are found. The catalytic cation is also shown (green). (*d*) HEPES binding environment in C54S SmeCOSe (PDB entry 7ptj).

The inhibition of the phosphatase superfamily by secondary products (sulfate and phosphate) has been well documented (Farooqui & Hanson, 1987 ▸; Ueki *et al.*, 1995 ▸; Scott & Spencer, 1968 ▸). Sulfate slightly decreased the activity of the partially purified *Aspergillus* and *Penicillium* COSe enzymes (Scott & Spencer, 1968 ▸; Segel & Johnson, 1963 ▸), but was reported not to inhibit *Pseudomonas* COSe (up to 30 m*M*; Takebe, 1961 ▸) or SmeCOSe (up to 70 m*M*; Sánchez-Romero & Olguin, 2015 ▸). Based on other studies showing the necessity of high sulfate concentrations to inhibit hydrolytic sulfatases (Hanson *et al.*, 2004 ▸), we used concentrations of sulfate of up to 200 m*M* to conduct TSA experiments with SmeCOSe, showing a 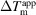 of 0.8°C. On the other hand, only higher concentrations of ammonium sulfate (up to 500 m*M*) produced a slight decrease (a 15% decrease) in SmeCOSe activity. A sulfate molecule could be modeled into the catalytic center of the WT SmeCOSe structure (Figs. 3 ▸ *a* and 4 ▸ *a*), most likely arising from the high concentrations of sulfate used in the crystallization conditions (1 *M* ammonium sulfate; Supplementary Fig. S1). Besides FGly54, our sulfate-bound structure confirms the involvement of His201 and Lys309 in sulfate binding (Fig. 4 ▸ *a*), which are proposed to be the acid/base catalytic residues (van Loo *et al.*, 2018 ▸), in agreement with other members of this superfamily (Hanson *et al.*, 2004 ▸; Jonas & Hollfelder, 2009 ▸). In the four chains modeled in this structure, Asn75 is at a distance between 2.8 and 3.1 Å from the sulfate ion; this residue occupies similar positions to Asn73 in *Rhizobium leguminosarum* PMH (Jonas *et al.*, 2008 ▸) and Arg166 in *E. coli* AP (Holtz & Kantrowitz, 1999 ▸), with the latter being reported to coordinate the substrate, the intermediate and the products of the reaction (Holtz & Kantrowitz, 1999 ▸). Based on mutational studies, and on the similarity to the arylsulfatase from *Pseudomonas aeruginosa* (Boltes *et al.*, 2001 ▸), Lys102 was suggested to complete the catalytic center of SmeCOSe by binding to the nonbridging O atoms of the sulfate moiety (van Loo *et al.*, 2018 ▸). However, our structures do not support this hypothesis, since Lys102 is at a distance of more than 4.0 Å in our sulfate-bound and HEPES-bound structures. Nonetheless, Lys102 is at a binding distance to FGly54 in this structure (Supplementary Fig. S8*a* ) and might assist during FGly regeneration (see below). Finally, whereas the sulfate moiety was hypothe­sized to interact with the cation of SmeCOSe (van Loo *et al.*, 2018 ▸), no direct interaction is observed between the metal ion and sulfate in the WT sulfate-bound structure (>3.3 Å) or in the HEPES-bound structure (>3.18 Å). In this sense, the interaction of phosphate or sulfate with the catalytic cation has been shown in other members of the AP superfamily such as alkaline phosphatase (Holtz *et al.*, 2000 ▸) and the WT aryl­sulfatase from *P. aeruginosa* (Boltes *et al.*, 2001 ▸). However, mutants of the latter (Miton *et al.*, 2018 ▸) and of the putative arylsulfatase from *E. coli* (PDB entry 3ed4; New York SGX Research Center for Structural Genomics, unpublished work) did not show this interaction.

#### 3.3.2. Choline binding

The product of the reaction, choline, has previously been shown to inhibit SmeCOSe (Sánchez-Romero & Olguin, 2015 ▸; van Loo *et al.*, 2018 ▸) and *Pseudomonas* COSe (Lucas *et al.*, 1972 ▸). TSA experiments showed an increase in the 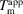 of WT and C54S SmeCOSe in the presence of choline (see above). Co-crystallization experiments of WT SmeCOSe with choline allowed the identification of choline molecules in the catalytic center of WT SmeCOSe (Figs. 3 ▸ *b* and 4 ▸ *b*; PDB entry 6g60). Despite the resolution of this structure, the densities present at the catalytic center suggested two ambiguous different orientations for choline. We thus decided to co-crystallize the C54S mutant with choline for comparison (PDB entry 7pth); the latter structure allowed a more reliable fitting of the choline moiety into the catalytic cleft (Figs. 3 ▸ *c* and 4 ▸ *c*). As previously mentioned, the choline-bound structures of the WT and the C54S mutant belonged to different space groups (Table 1 ▸), although no appreciable structural differences were observed between them due to different crystal packing (Supplementary Fig. S7); in particular, the regions containing residues important for the activity of SmeCOSe were highly similar.

Our structures clearly illustrate a deep L-shaped hollow into which the ligand needs to diffuse to reach the catalytic pocket (Supplementary Fig. S9). This tunnel is constituted by a considerable concentration of aromatic residues (Tyr123, Trp129, Trp142, Trp143, His145 and His201) and also residues from the C-terminal region of an adjacent subunit (Asn495–Leu499; Supplementary Fig. S6). This entrance has previously been described in detail, and an important inter-subunit hydrogen bond was suggested to connect the protomers at the substrate entrance (Asn146–Asp500; see below; van Loo *et al.*, 2018 ▸). Choline is placed at the bottom of the ‘aromatic tunnel’ both in the WT and the C54S mutant structures. In all cases, the quaternary ammonium (quat) moiety of choline appears to be sandwiched at approximately 3.5 Å from the aromatic rings of Trp129 and His145 (Figs. 4 ▸ *b* and 4 ▸ *c*). During the review process of this work, Li and coworkers published a must-read article on sulfatases, in which docking studies of the SmeCOSe structure with COS suggested the importance of Trp129 and His145 in choline binding (Li *et al.*, 2021 ▸). Besides Trp129 and His145, other residues within a 5 Å distance of the N atom of the quat group are Tyr123, Trp143 and Tyr144, and also Leu499 from an adjacent subunit; the carbonyl group of the peptide bond of Trp143 and the hydroxyl group of Tyr123 are placed at distances of 3.5–4.0 Å from the quat N atom. All of these residues are highly conserved among different putative choline sulfatases (Supplementary Fig. S10; see also van Loo *et al.*, 2018 ▸). The interaction with Trp129 and His145 resembles the common binding mode observed in other choline-binding proteins (CBPs) and is explained by interactions of the quat group with π electrons of aromatic amino-acid side chains (Schiefner, Breed *et al.*, 2004 ▸; Schiefner, Holtmann *et al.*, 2004 ▸; Horn *et al.*, 2006 ▸; Wolters *et al.*, 2010 ▸; Fernández-Tornero *et al.*, 2002 ▸). Comparison with other CBPs reveals that CbpJ from *Streptococcus pneumoniae* presents a conserved choline-binding hairpin (GW-*X*_6_-WYY motif, where the underlined residues correspond to the binding residues; see, for example, PDB entry 6jyx; Xu *et al.*, 2019 ▸). A *BLAST*/phylogenetic analysis of SmeCOSe reveals that it shows a similar conserved GW-*X*_13_-WYHN choline-binding hairpin motif (Supplementary Fig. S10), where the underlined residues correspond to Trp129 and His145 in SmeCOSe. Surprisingly, we found that a similar binding strategy is adopted by the phosphorylcholine esterase domain of the virulence factor CBP-E from *S. pneumoniae*, a member of the metallo-β-lactamase superfamily with a similar bimetallic center to that appearing in various members of the AP superfamily (Garau *et al.*, 2005 ▸). This observation further supports the phylogenetic relationships proposed between the members of these superfamilies (Baier & Tokuriki, 2014 ▸; van Loo, Bayer *et al.*, 2019 ▸), providing a new example of convergent adaptation to similar substrates (choline-sulfate and choline-phosphate) after divergent evolution of the ancient AP and metallo-β-lactam­ase superfamilies.

Comparison of the structures of the WT and the C54S mutant shows that despite conservation of the position of the quat moiety in both structures, choline could be modeled in several orientations in the different chains in both structures (Figs. 4 ▸ *b* and 4 ▸ *c*, respectively). Interactions of the hydroxyl moiety of choline can be envisioned with Asn75 or Asp386 (at less than 3.0 Å; chains *A*, *B* and *C* of PDB entry 6g60) or with Lys309 and His201 (at 3.0 and 3.5 Å, respectively; chain *D* of PDB entry 6g60) (Figs. 4 ▸ *b* and 4 ▸ *c*). This molecule could be more reliably fitted in the C54S mutant structure (Figs. 3 ▸ *b* and 3 ▸ *c*). The hydroxyl moiety of choline in this structure is 3.0–3.5 Å from Lys309, but also makes various water-mediated interactions with Glu386, Asn75 and the peptide bond of Ser54. Asp386 further occupied different positions in the C54S mutant structure (Fig. 4 ▸ *c*). Alternative ligand conformations have previously been proposed for human arylsulfatase (PDB entry 1e2s), in which the ligand hydroxyl group also points towards an Asp residue (von Bülow *et al.*, 2001 ▸). Whereas Asp386 was shown to be key in the activity of SmeCOSe by mutational analysis, none of our structures support the previous hypothesis on the binding of this residue to the quat moiety of choline (van Loo *et al.*, 2018 ▸); substrate binding via Asp386 is unlikely to occur since the hydroxyl moiety of the substrate would be too far from this residue (Figs. 4 ▸ *b*, 4 ▸ *c*, 4 ▸ *d* and Supplementary Fig. S11). However, the alternative conformations observed for Asp386 and choline in the choline-bound WT structure (Figs. 4 ▸ *b* and 4 ▸ *c*), together with the plausible interaction observed between choline and Asp386 through the hydroxyl moiety (Fig. 4 ▸ *b*) or through water-mediated interactions (see below), suggest a plausible transient interaction among them, which is most likely necessary to stabilize choline after substrate hydrolysis or to assist during product release.

#### 3.3.3. Alkyl substrate-like binding

Whereas the binding of COS to the C54S mutant was confirmed by TSA experiments (Fig. 2 ▸ *c*), we were unsuccessful in obtaining a structure showing this substrate in the catalytic center. Serendipitously, we were able to obtain a HEPES-bound structure of C54S SmeCOSe due to the change of the crystallization buffer during the crystal-improvement experiments (PDB entry 7ptj). As in the choline-bound WT structure, high variability could be observed in the different subunits of the HEPES-bound structure, with the fitting of chain *A* being the most reliable (Fig. 3 ▸ *d*). This variability might arise from the presence of sulfate in the crystallization solution, which is likely to produce partial occupation of the catalytic centers in the different subunits. TSA experiments showed an increase in the 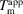 for the C54S species with HEPES and other sulfate-containing molecules (Supplementary Fig. S5).

The sulfate moiety of HEPES occupies approximately the same positions as in the sulfate-bound structure, and it is also at a distance of less than 3.0 Å from Ser54, Asn75, His201 and Lys309 (Figs. 4 ▸ *d* and 5 ▸). The piperazine core of HEPES is accommodated between the Trp129–His145 pair, as is the choline quat moiety: the N atoms of both species occupy nearly the same position (Figs. 4 ▸ *d* and 5 ▸). Additional polar interactions with the hydroxyl moiety of the alkyl side chain of HEPES appear, such as that with the peptide bond of residues His145 or Asn146 (Fig. 4 ▸ *d*). The Asn146–Asp500 interaction is in an open conformation in this structure (Fig. 6 ▸). Since no clear density appears for Asp500, this residue is most likely to be responsible for conducting the opening/closure of the aromatic tunnel, turning it into the gatekeeper (see below).

### 3.4. Mechanistic implications for the activity of SmeCOSe

Our WT and C54S SmeCOSe structures allow a rigorous analysis of the ligand-binding mode of SmeCOSe. Half a century ago, an overall reaction mode for COSe hydrolysis was hypothesized based on the different competitive or uncompetitive characters of different ligands, suggesting conformational changes in the catalytic center of COSe to accept the quat moiety (Lucas *et al.*, 1972 ▸). Based on mutational and structural analysis, different key residues for COSe catalysis have previously been proposed (van Loo *et al.*, 2018 ▸). Very recent work has proposed plausible residues involved in substrate interaction and the significance of the C-terminal region in the substrate specificity of various sulfatases, including SmeCOSe (Li *et al.*, 2021 ▸), with theoretical docking results which partly reflect the results presented in this work. However, our structures provide clues to determinants governing the catalysis of these enzymes that differ from those previously proposed.

#### 3.4.1. Substrate entrance is controlled by a gatekeeper element

**Figure 5:**
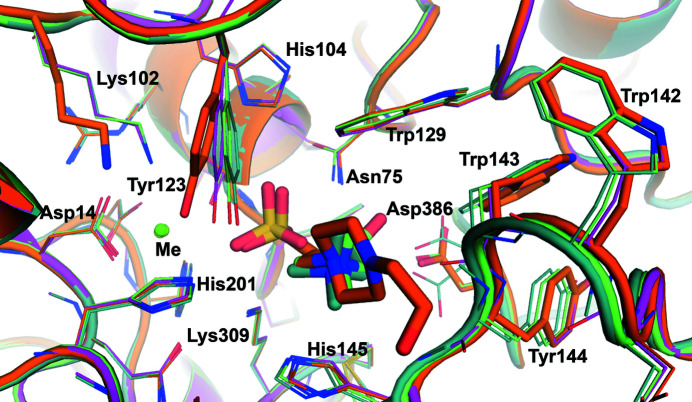
Superposition of chain *A* of the different structures, showing the molecules bound in the catalytic cleft of WT and C54S mutant SmeCOSe species. Sulfate-bound WT SmeCOSe (PDB entry 6g5z) is shown in pink tones, choline-bound WT SmeCOSe (PDB entry 6g60) in dark green tones, choline-bound C54S SmeCOSe (PDB entry 7pth) in light green tones and choline-bound C54S SmeCOSe (PDB entry 7ptj) in orange tones. The catalytic metal cation (Me) is also shown in green.

**Figure 6:**
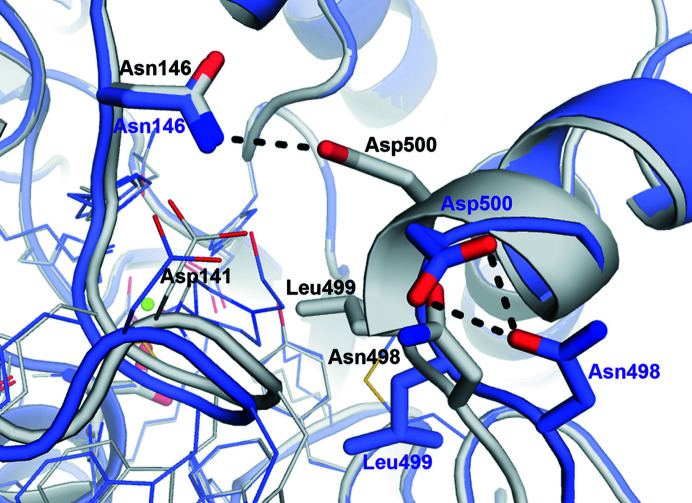
Open and closed conformations of SmeCOSe showing the rearrangement of Asn146–Asp500 (inter-subunit) to Asn498–Asp500 (intra-subunit). PDB entry 7ptj (chain *A*, blue tones; chain *B*, purple tones) shows the open conformation, whereas PDB entry 6g60 (chain *A*, yellow tones; chain *B*, dark yellow tones) shows the closed conformation. The Asn146–Asp500 (closed conformation) and Asn498–Asp500 (open conformation) interactions are highlighted in black. The direction of the movement of Leu499 is also highlighted.

**Figure 7:**
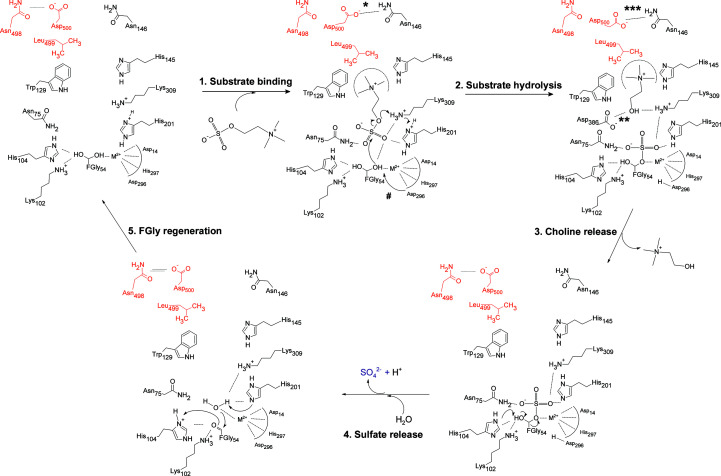
Proposed reaction mechanism for hydrolysis of choline-*O*-sulfate by SmeCOSe based on structural models. Residues participating in the catalytic center of SmeCOSe from a different subunit appear in red. (1) Substrate binding is likely to occur concomitantly with closure of the Asp500–Asn146 hydrogen bond (*), producing the rearrangement of Leu499 towards the catalytic site. The sulfate moiety is bound to Asn75, His201 and Lys309, whereas the choline moiety is ‘clamped’ between His145 and Trp129. (2) Activation of the diol of FGly54 by His201 would allow an S_N_2 attack, and cleavage of choline-sulfate, followed by protonation of the choline alkoxylate moiety by Lys309. (3) Lys309 can be reprotonated, probably by the same proton bound to His201. Rearrangement of choline into the catalytic cleft by direct or water-mediated contacts with Asp386 (**) is suggested by our structures, which might unleash the aperture of gating residues (***), allowing choline release. (4) Deprotonation of the covalent FGly sulfate intermediate by His104 allows a rearrangement producing the FGly aldehyde and release of sulfate, accompanied by the entrance of water into the catalytic cleft. (5) Rehydration of the FGly aldehyde is expected to occur by this metal-activated water molecule as proposed previously (Hanson *et al.*, 2004 ▸). This water molecule is also at a binding distance from His201/Lys309. By comparison with other sulfatase structures, the residue suggested to deprotonate the water molecule would be Asp396 (van Loo, Berry *et al.*, 2019 ▸). However, His297 (also one of the metal-bound residues) is at a shorter binding distance from the water molecule in our structures (#). His201 is also at a binding distance from this water molecule in the choline-bound C54S mutant structure.

A highly conserved inter-subunit hydrogen bond (Asn146–Asp500; Supplementary Fig. S10) has been reported to ‘control’ the entrance to the active site (van Loo *et al.*, 2018 ▸). Our structures show different ‘open’ (PDB entries 6g5z, 6g60 and 7ptj) or ‘closed’ (PDB entries 6g60 and 7pth) conformations for these residues (Fig. 7 ▸). An additional intra-subunit hydrogen bond (Asn498–Asp500) further suggests a dynamic rearrangement among Asn146–Asp500–Asn498, suggesting Asp500 to be the gatekeeper in SmeCOSe (Fig. 6 ▸). On the other hand, Asn498 is not totally conserved among different COSes (Supplementary Fig. S9). Several zones are affected upon such a rearrangement, specifically the Trp129–Asn146 region (Figs. 5 ▸ and 6 ▸) and also Leu499, which shifts towards the catalytic center in the ‘closed’ conformation. This residue is displaced approximately 5 Å between the closed and open conformations (Fig. 6 ▸) and is situated less than 3.5 Å from the methyl groups of the quat moiety of choline (Supplementary Fig. S12). Trp129 also interacts with Asn495 from the C-tail of an adjacent protomer (Supplementary Fig. S12), which contains the above-described Asn498 and Asp500 residues. The Asp481–Arg494 loop preceding the C-terminal region also provides additional inter-subunit contacts, forming a cross-section with the same segment of an adjacent subunit. In particularly, the salt bridge between Asp481 and Arg494 is totally conserved among different COSes (Supplementary Fig. S10) and most likely ‘fastens’ both ends of this segment with additional salt bridges (Supplementary Fig. S12). Thus, besides the importance of the C-termini in the previously proposed oligomerization of SmeCOSe (van Loo *et al.*, 2018 ▸), a network of interactions is present connecting the choline-binding residues Trp129–Asn146 to the C-termini of the enzyme and an additional protomer. Positioning of the ligand between the Trp129/His145 pair might represent the chemical signal that unleashes the binding of Asn146–Asp500 and the rearrangement of Leu499 towards the catalytic cleft (Fig. 6 ▸ and Supplementary Fig. S12). Structural superposition with other representative members of the family shows that the C-tail is unique to SmeCOSe, and as far as we know SmCOSe provides the first snapshots of an AP member that shows dynamic conformational gating (Gora *et al.*, 2013 ▸). Molecular-dynamics simulations carried out with the sulfate-bound WT SmeCOSe structure using the *CABS-flex* server 2.0 (Kuriata *et al.*, 2018 ▸) support plausible large fluctuations in the SmeCOSe structure, including its C-tail (Supplementary Fig. S13). This feature, together with the substrate promiscuity and proficiency of SmeCOSe (van Loo *et al.*, 2018 ▸), might turn this enzyme into a significant challenge to engineering protein dynamics, both theoretically and experimentally (Gardner *et al.*, 2020 ▸). From the mechanistic point of view, the open/closed conformations of SmeCOSe might greatly condition its kinetics, even turning this movement into the rate-limiting step. Our results explain why the truncation of the last 12 residues at the C-termini of SmeCOSe resulted in a much higher impact on the enzymatic activity with the natural substrate than with pNPS, proving the role of Leu499 in assisting in the correct positioning of the quat moiety of choline (Fig. 6 ▸ and Supplementary Fig. S12), but it is not necessarily required for pNPS positioning. Leu499 does not interact with the cyclic moiety of the HEPES molecule (Fig. 4 ▸ *d*), which is analogous to the cyclic moiety of pNPS.

#### 3.4.2. Reaction mechanism of SmeCOSe

Despite the high conservation of the catalytic centers of different sulfatases belonging to the AP superfamily (van Loo, Bayer *et al.*, 2019 ▸; Boltes *et al.*, 2001 ▸; Jonas & Hollfelder, 2009 ▸; Hanson *et al.*, 2004 ▸), different roles have been proposed for the residues involved in substrate catalysis over the years, for example, in PMHs and arylsulfatases (van Loo, Bayer *et al.*, 2019 ▸; van Loo *et al.*, 2010 ▸; van Loo, Berry *et al.*, 2019 ▸; Miton *et al.*, 2018 ▸; Luo *et al.*, 2012 ▸), exo-2*S*-ι-carrageenan S1 sulfatase (Hettle *et al.*, 2019 ▸), iduronate-2-sulfatase (Demydchuk *et al.*, 2017 ▸), chondroitin sulfate/dermatan sulfate 4-*O*-sulfatases (Wang *et al.*, 2019 ▸) or other sulfatases (van Loo, Bayer *et al.*, 2019 ▸; Li *et al.*, 2021 ▸) (Supplementary Table S1). Specifically, two highly conserved His/Lys pairs (namely His^*A*^/Lys^*A*^ and His^*B*^/Lys^*B*^ according to the nomenclature used previously; Hanson *et al.*, 2004 ▸) have been proposed to show different specific functions despite their general involvement in ligand binding or in FGly regeneration (Boltes *et al.*, 2001 ▸). Nevertheless, our SmeCOSe structures allow a rigorous analysis of the plausible ligand-binding mode, providing an alternative scenario to that proposed previously (van Loo *et al.*, 2018 ▸). Superposition of chains *A* of the four structures (Fig. 5 ▸) show differences in Tyr123 (of up to 2.0 Å), Trp142, Trp143, Tyr144 (of up to 1 Å) and Asp386 (which shows significant different conformations in the WT choline-bound structure). The largest shift of Tyr123 occurs in the HEPES-bound structure and might be attributed to the bulkier piperazine ring compared with the methyl groups of choline. Lys102 (representing Lys^*A*^ in other sulfatases; Hanson *et al.*, 2004 ▸) is also displaced, but only in the HEPES-bound C54S structure (Fig. 5 ▸). Thus, besides the opening/closing movement and the rearrangement of Leu499, the repositioning of different residues of the catalytic center is ascertained, further supporting the plasticity of the catalytic cleft of SmeCOSe.

**Figure 8:**
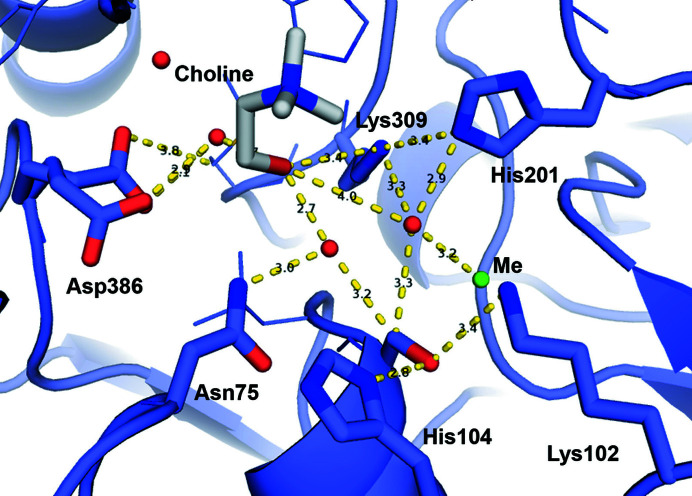
Environment of the water situated near the metal cation in the choline-bound C54S SmeCOSe structure (PDB entry 7pth chain *A*), which might be used to regenerate the FGly residue. The catalytic cation (Me) is also shown in green.

By similarity to the binding observed for sulfate, choline and HEPES (Fig. 4 ▸), the sulfate moiety of COS is expected to bind FGly54, Asn75, His201 and Lys309 after substrate entrance, whereas the quat moiety would be placed between Trp129 and His145 (Fig. 7 ▸ and Supplementary Fig. S11). The counterparts of the Asn75 residues in different sulfatases are also reported to assist sulfate binding (Hanson *et al.*, 2004 ▸; van Loo, Bayer *et al.*, 2019 ▸), although this residue was not included in the previously proposed reaction mechanism of SmeCOSe (van Loo *et al.*, 2018 ▸). Lys102 and His104 (the counterparts of His^*A*^/Lys^*A*^; Hanson *et al.*, 2004 ▸) are at binding distances from FGly and Ser54 (Fig. 8 ▸). His201 and Lys309 (the counterparts of His^*B*^/Lys^*B*^; Hanson *et al.*, 2004 ▸; van Loo, Bayer *et al.*, 2019 ▸, were proposed to stabilize and facilitate the breakage of the transition state with concomitant desulfation of the substrate, protonating the leaving hydroxyl group (or amine, depending on the substrate; Sidhu *et al.*, 2014 ▸). The most accepted reaction mechanism for FGly suggests that the substrate is desulfated concomitantly with sulfonylation of the enzyme through the FGly diol residue, which is then desulfated in turn (Appel & Bertozzi, 2015 ▸; Hanson *et al.*, 2004 ▸; Fig. 7 ▸). Experimental evidence showed that catalysis by SmeCOSe proceeds through S—O cleavage, in contrast to the expected C—O attack, according to the classical classification of sulfatases (van Loo *et al.*, 2018 ▸). Thus, an S_N_2 substitution is expected for SmeCOSe, as proposed for other arylsulfatases (Boltes *et al.*, 2001 ▸; von Bülow *et al.*, 2001 ▸), where the FGly diol would be responsible for substrate hydrolysis. A conserved metal-bound Asp has been proposed to activate the FGly diol residue in different AP superfamily members (Asp^*A*^; Boltes *et al.*, 2001 ▸; Hanson *et al.*, 2004 ▸); the counterpart residue in SmeCOSe is Asp296 (Fig. 7 ▸). However, His297 is also at a binding distance from the FGly diol and might be a candidate for a similar mechanism. After substrate hydrolysis, the hydroxylate moiety of choline is expected to be protonated due to its high p*K*_a_; since our structures show Lys309 at a binding distance from the hydroxyl moiety of choline (but not His201), its side chain is likely to be responsible for protonation after substrate hydrolysis (Fig. 7 ▸). Reprotonation of Lys309 is plausible through the protonated His201, since both residues appear at <3.5 Å. Rearrangement of choline into the catalytic cleft after hydrolysis seems to unleash the opening of the gatekeeper Asp500, allowing the release of this reaction product.

Following the release of choline, His104 would be responsible for deprotonating the hydroxyl moiety of FGly that is not covalently bound to sulfate, allowing sulfate release (Fig. 7 ▸). FGly needs then to be hydrated to a geminal diol, it being accepted that a water molecule reconstitutes the FGly diol after substrate hydrolysis (Appel & Bertozzi, 2015 ▸; Hanson *et al.*, 2004 ▸). Our choline-bound C54S SmeCOSe structure shows a water molecule at a binding distance from the cation and Ser54 (Fig. 8 ▸), in approximately the same position at which the germinal diol of FGly appears in the WT structure; this water molecule is also at a binding distance from His201 and Lys309. Lys102 and His104 are at a binding distance from Ser54 in this structure (Fig. 8 ▸) and also in both WT structures (although Lys102 is displaced in the choline-bound C54S mutant). Based on the importance of Lys102 in the activity of SmeCOSe that has been shown previously (van Loo *et al.*, 2018 ▸), we cannot totally rule out a transient interaction with the sulfate moiety of the substrate or the reaction product, and this cannot be ascertained from our sulfate- and HEPES-bound structures. On the other hand, Lys102 might be involved in FGly positioning and its mutation could also cause an impairment of activity. Since His201 is at a binding distance from the plausible regenerating water molecule, it is plausible that His201 retrieves this proton (Fig. 7 ▸), which would be used to reprotonate Lys309 in the next reaction cycle. In addition, the nucleophilic attack of this water molecule allows the deprotonation of His104 to regenerate the FGly diol.

Different promiscuous enzymes rely on water-bridged ligand interactions for their differential substrate binding, such as RNAses (Ivanov *et al.*, 2019 ▸), *N*-succinyl-amino-acid racemases (Martínez-Rodríguez *et al.*, 2020 ▸) and cytochrome P450 (Madrona *et al.*, 2013 ▸). This feature has also been observed for promiscuous solute-binding proteins (Clifton & Jackson, 2016 ▸; Matsuoka *et al.*, 2015 ▸; Camara-Artigas *et al.*, 2016 ▸), and in fact other AP superfamily members present water-mediated ligand interactions, such as the promiscuous ectonucleotidase NPP1 (Namasivayam *et al.*, 2017 ▸; Dennis *et al.*, 2020 ▸), endo-4*S*-ι-carrageenan sulfatase (Hettle *et al.*, 2018 ▸) and *N*-acetylgalactosamine-6-*O*-sulfatase (Ndeh *et al.*, 2020 ▸). Our structural models clearly support the involvement of water-mediated interactions assisting choline positioning after hydrolysis, where Lys309, Asp386 and Asn75 might assist in leaving-group stabilization (Figs. 4 ▸, 7 ▸ and 8 ▸). The net of water molecules around choline (PDB entries 6g60 and 7pth; Fig. 8 ▸) also connects choline to the peptide chain of Asn146, which is one of the residues forming the gate of SmeCOSe. The dynamic character observed for choline might ‘switch off’ the Asn146–Asp500–Asn498 gate, turning the enzyme back to the open position. Since the loop containing Asn498 and Asp500 is interconnected to Trp129 thorough Asn495 belonging to the C-tail of an adjacent protomer (Supplementary Fig. S12), it is arguable that changes in choline positioning after substrate hydrolysis could affect (i) the network of water molecules connecting to Asn498 and (ii) the Trp129–Asn495 interaction, unleashing the opening/closure of the enzyme as a result of subtle changes in ligand binding.

## 4. Concluding remarks

In conclusion, here we report the first crystal structures of ligand-bound WT and C54S SmeCOSe at below 1.9 Å resolution. Structural analyses allow the different molecular mechanisms governing SmeCOSe catalysis to be deciphered. Despite SmeCOSe showing a catalytic proficiency (*k*_cat_/*K*_m_/*k*_1_) for COS hydrolysis of 1.7 × 10^25^ *M* ^−1^, its catalytic efficiency (*k*_cat_/*K*_m_) for this substrate is of the order of 10^5^ *M* (van Loo *et al.*, 2018 ▸), representing the value for an ‘average enzyme’ (Bar-Even *et al.*, 2011 ▸). The large differences that are observed could be explained if the reaction mechanism of COSe is diffusion-controlled, because of the opening/closing movement described here. The structural information also argues against the proposed role for conformational dynamics in promoting the enzymatic catalytic proficiency of an enzyme (Miller & Wolfenden, 2002 ▸): the ‘open’ SmeCOSe conformation (Fig. 6 ▸) allows substrate access and product release from the catalytic cleft, whereas the closed conformation (Fig. 6 ▸) maximizes contacts with the substrate in its transition state, as suggested by the displacement of Leu499 and its positioning towards the quat moiety of choline. Since members of the AP superfamily have been profoundly studied in attempts to understand evolutionary and mechanistical aspects (Barrozo *et al.*, 2015 ▸; Pabis & Kamerlin, 2016 ▸; van Loo, Bayer *et al.*, 2019 ▸; Mohamed & Hollfelder, 2013 ▸; Pabis *et al.*, 2016 ▸; Duarte *et al.*, 2013 ▸), the structural information provided here supporting the conformational flexibility of the catalytic cleft of SmeCOSe might help to obtain insights into the substrate promiscuity and proficiency of the AP superfamily.

## 5. Related literature

The following reference is cited in the supporting information for this article: Crooks *et al.* (2004 ▸).

## Supplementary Material

PDB reference: choline-*O*-sulfatase, wild type, bound to sulfate, 6g5z

PDB reference: wild type, bound to choline, 6g60

PDB reference: C54S mutant, bound to choline, 7pth

PDB reference: C54S mutant, bound to HEPES, 7ptj

Supplementary Figures and Table. DOI: 10.1107/S2059798322003709/cb5132sup1.pdf

## Figures and Tables

**Table 1:** Data-collection and refinement statistics Values in parentheses are for the highest resolution shell.

	Choline-bound WT SmeCOSe	Sulfate-bound WT SmeCOSe	Choline-bound C54S SmeCOSe mutant	HEPES-bound C54S SmeCOSe mutant
PDB code	6g60	6g5z	7pth	7ptj
Data collection				
Space group	*C*121	*C*121	*P*12_1_1	*C*121
*a*, *b*, *c* (Å)	128.54, 206.90, 116.60	128.20, 206.5, 116.7	93.77, 103.97, 108.97	128.52, 207.01, 116.83
α, β, γ (°)	90, 110.17, 90	90, 110.28, 90	90, 104.037, 90	90, 110.29, 90
Resolution (Å)	56.78–1.84 (1.91–1.84)	19.98–1.98 (2.05–1.98)	45.49–1.85 (1.92–1.85)	75.25–2.10 (2.17–2.10)
*R*_merge_ (%)	3.03 (36.93)	2.58 (73.9)	9.02 (112.4)	6.37 (38.9)
〈*I*/σ(*I*)〉	13.14 (2.27)	12.98 (2.40)	9.85 (1.33)	8.57 (2.22)
Completeness (%)	95.01 (93.84)	99.85 (99.94)	93.15 (94.70)	94.90 (92.97)
Unique reflections	234059 (23094)	196583 (19594)	161208 (16354)	157655 (15403)
Multiplicity	1.8 (1.7)	2.0 (2.0)	4.6 (4.7)	1.9 (1.9)
Wilson *B* factor (Å^2^)	27.66	31.94	23.73	30.07
CC_1/2_	0.999 (0.77)	0.999 (0.86)	0.998 (0.703)	0.995 (0.756)
Refinement				
*R*_work_/*R*_free_ (%)	17.08/20.01	17.11/20.04	16.29/20.06	16.20/19.97
No. of atoms				
Total	17304	16978	19122	18795
Protein	16019	16043	16920	16716
Ligands	67	74	149	294
Solvent	1218	861	2143	1833
*B* factor (Å^2^)	34.03	41.83	28.46	34.14
R.m.s. deviations				
Bond lengths (Å)	0.010	0.006	0.007	0.008
Bond angles (°)	1.33	1.18	0.88	0.89
Ramachandran statistics (%)				
Favored	97.26	97.10	96.93	96.91
Outliers	0.15	0.15	0.15	0.15
