# Supplementary material for: Structural insights into choline-O-sulfatase reveal the molecular determinants for ligand binding
Source: Acta Crystallogr D Struct Biol. 2022 Apr 26;78(Pt 5):669–82. doi: 10.1107/S2059798322003709 (PMC9063841; doi:10.1107/S2059798322003709)
Supplement: Supplementary file 1 [file d-78-00669-sup1.pdf]

STRUCTURAL
BIOLOGY

Volume 78 (2022)

Supporting information for article:

Structural insights into choline-*O*-sulfatase reveal the molecular determinants for ligand binding

Jose Antonio Gavira, Ana Cámara-Artigas, Jose Luis Neira, Jesús M. Torres de Pinedo, Pilar Sánchez, Esperanza Ortega and Sergio Martinez-Rodríguez

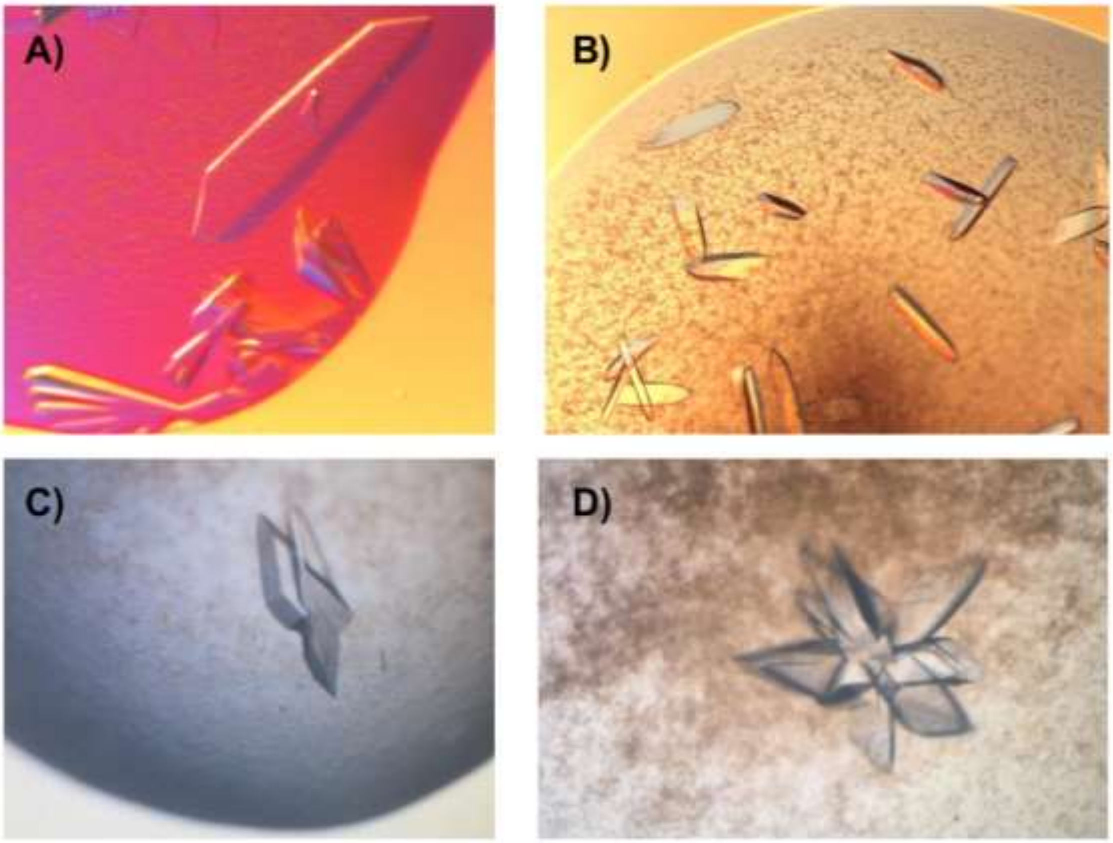

Figure S1. WT and C54S SmeCOSe crystals used for structural determination. A) WT SmeCOSe crystals grown in 1.0 M $LiSO_4$ 0.1 M Tris (pH 7.0) (PDB 6G5Z). B) WT SmeCOSe co-crystallized with choline, using 1.0 M $LiSO_4$ 0.1 M Tris (pH 7.0) (PDB 6G60). C) C54S SmeCOSe co-crystallized with choline using 0.2 M Sodium acetate trihydrate 0.1 M Tris (pH 8.5) 30% w/v Polyethylene glycol 4,000. D) C54S SmeCOSe crystals obtained using 1.5 M $LiSO_4$ 0.1M HEPES (pH 7.5).

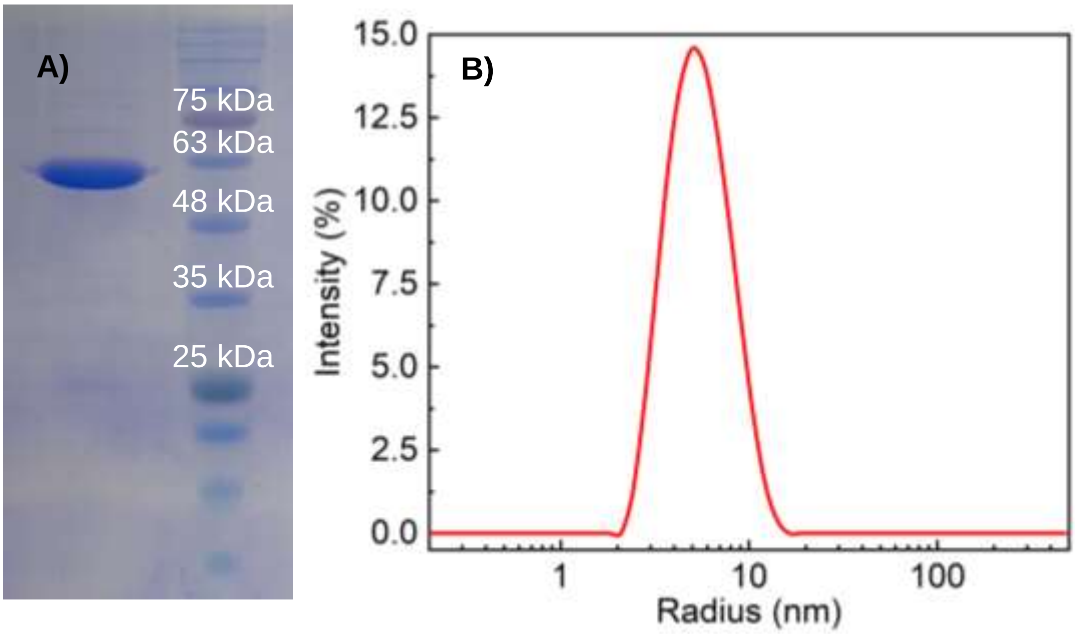

Figure S2. A) SDS-PAGE of purified recombinant SmeCOSe. B) Particle size distribution by intensity of the light scattered by 20 µM SmeCOSe in 20 mM Tris (pH 8.0) show a R_h value of 5.7±2.1 nm, which is characteristic of a globular protein of approximately 200kDa. No large aggregates were found.

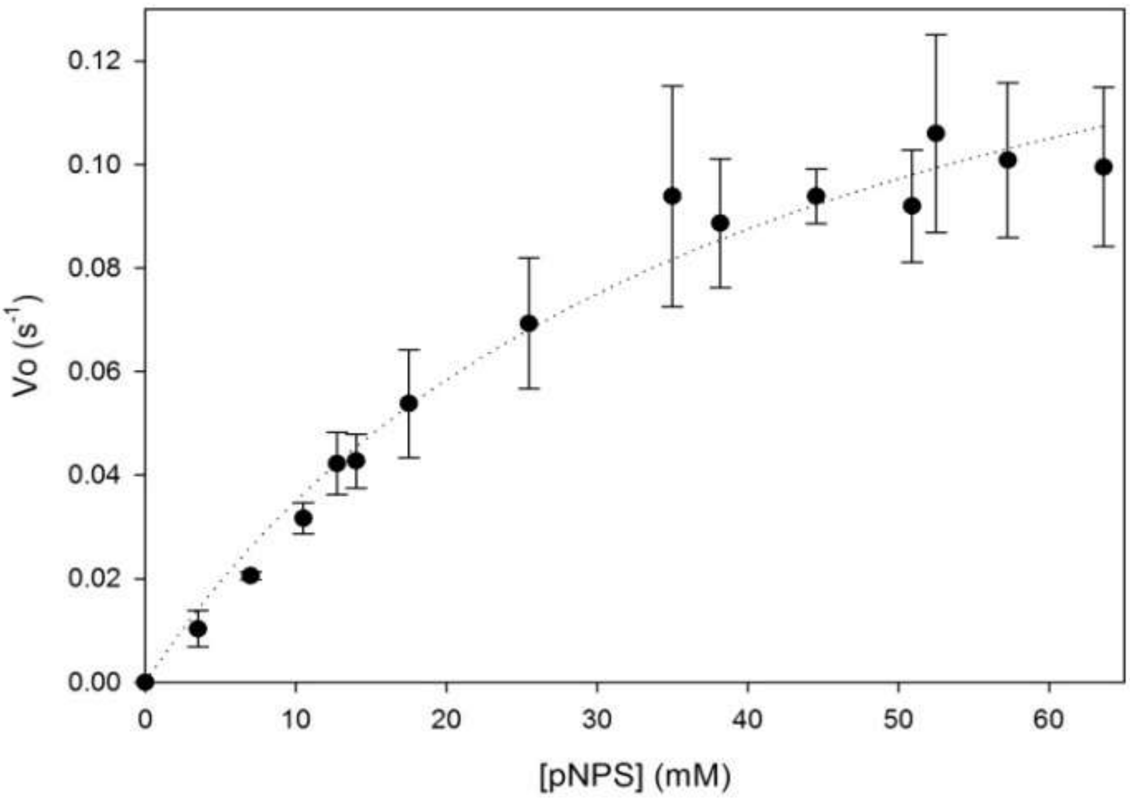

Figure S3. Kinetic experiments of SmeCOSe with pNPS conducted in 25 mM Tris (pH 7) at 37 ºC. A k_{cat} of 0.22±0.03 s^{-1} and a K_m of 45.7±12.5 mM were obtained for this substrate.

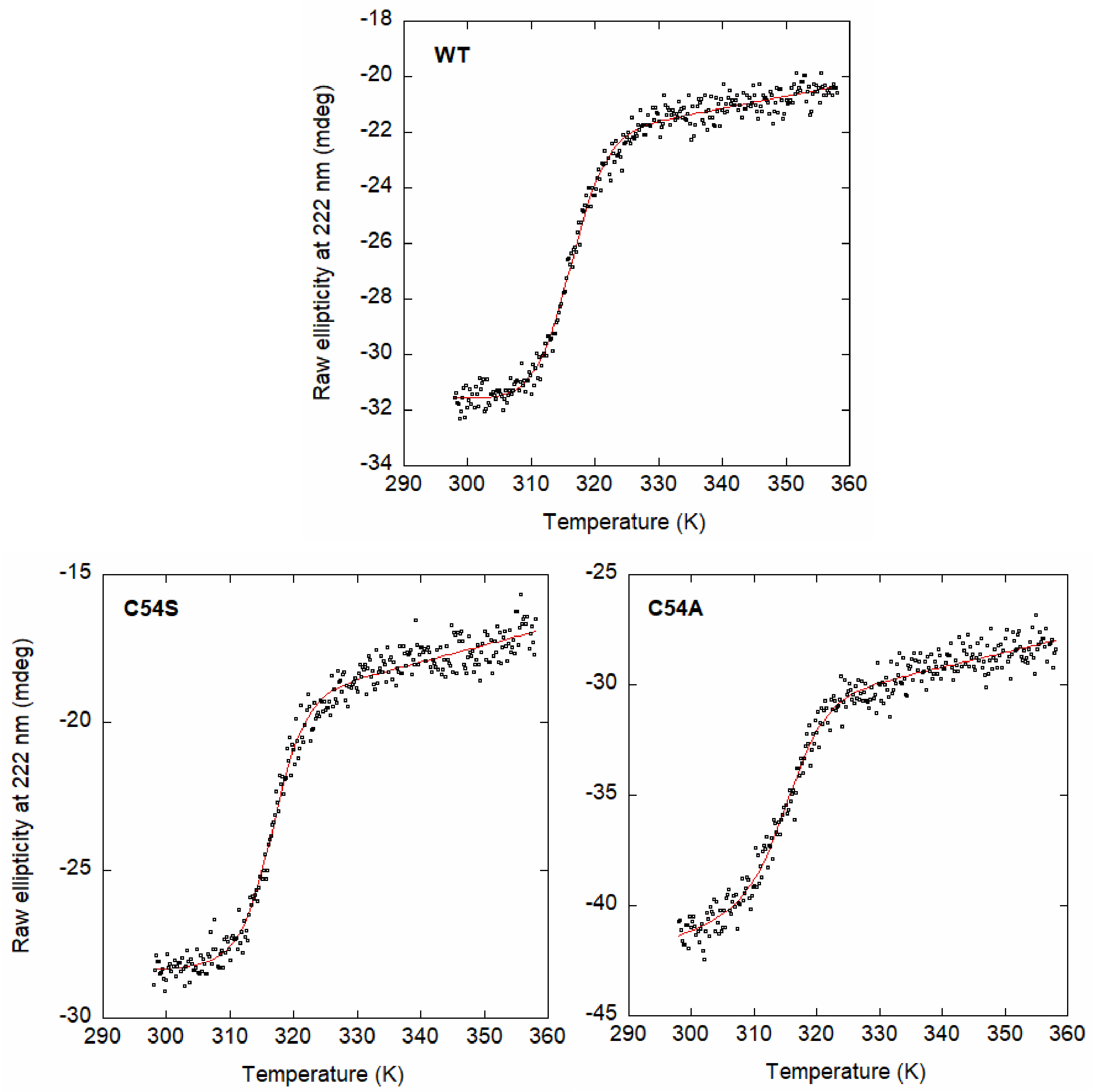

Figure S4. Fitting of Raw data of Far-UV CD denaturation profiles of WT (A), C54A mutant (B) and C54S mutant SmeCOSe species. Thermal midpoints were estimated to be 315.9 (WT), 316.5 (C54S) and 315.2 K (C54A), respectively. Fitting to the two-state equation (considering the oligomeric nature of the protein) is shown as a red line.

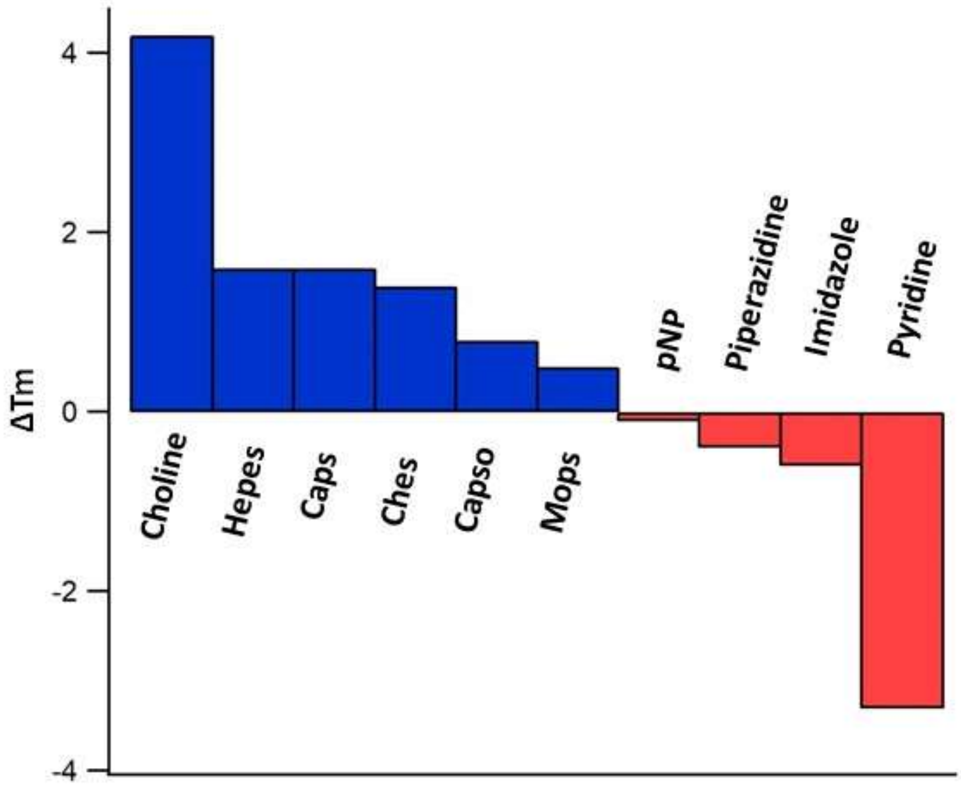

Figure S5. Differences observed (ΔT_m) in the apparent thermal midpoints, T_m^{app}, of SmeCOSe C54S in the presence of different compounds.

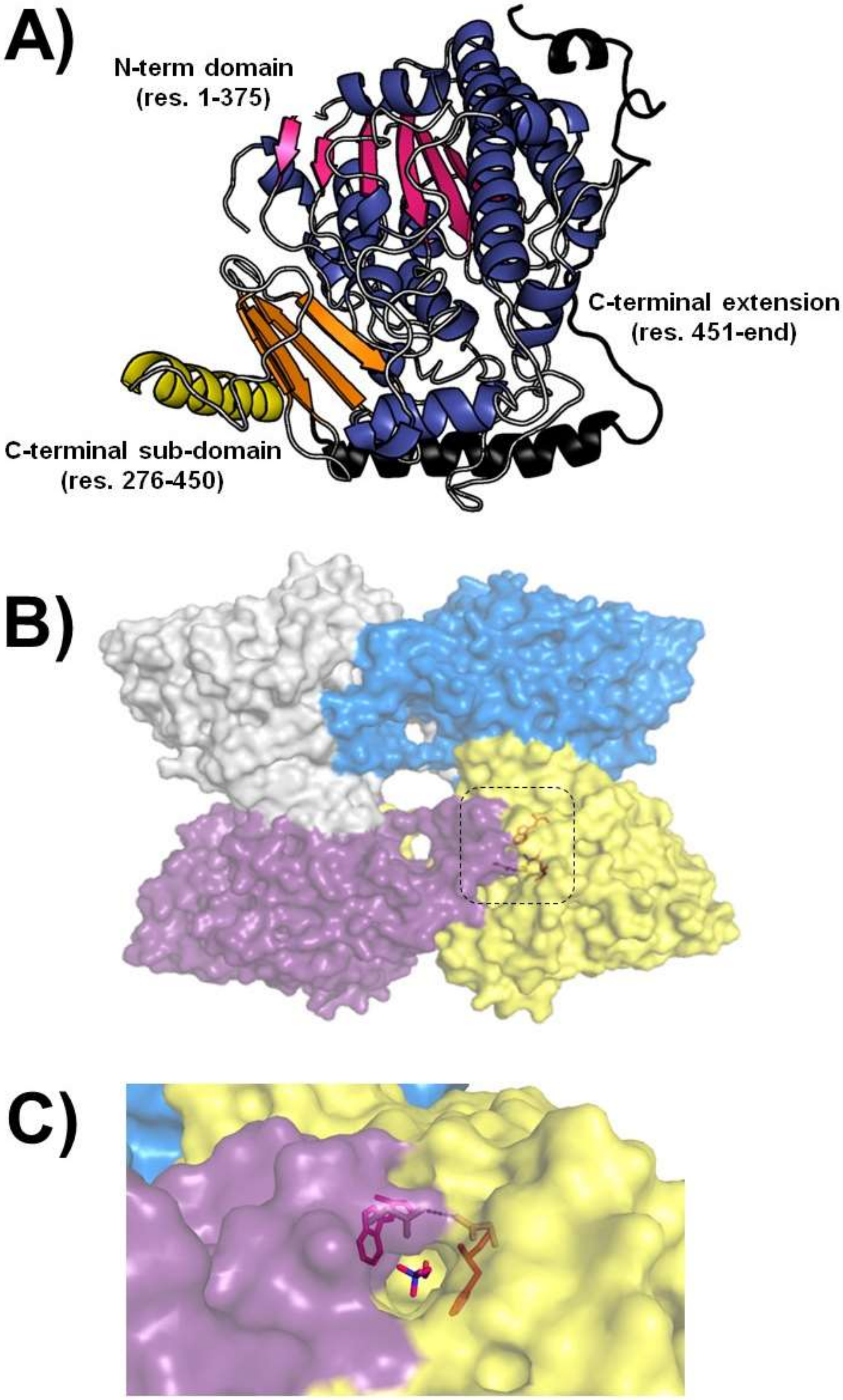

Figure S6. A) Monomeric arrangement showing the two main structural elements common to the alkaline phosphatase superfamily: a large N-terminal domain (α-β-α sandwich, blue and magenta) and a smaller C-terminal sub-domain (α/β 2-layer sandwich, orange and yellow). The C-terminal extension containing two alpha-helices, which embraces the N-terminal domain is also shown (black). B) Tetrameric arrangement of SmeCOSe, highlighting substrate entrance. C) Upper-sight of ligand-binding site showing the position of “clamped” choline by residues Trp129 and His145.

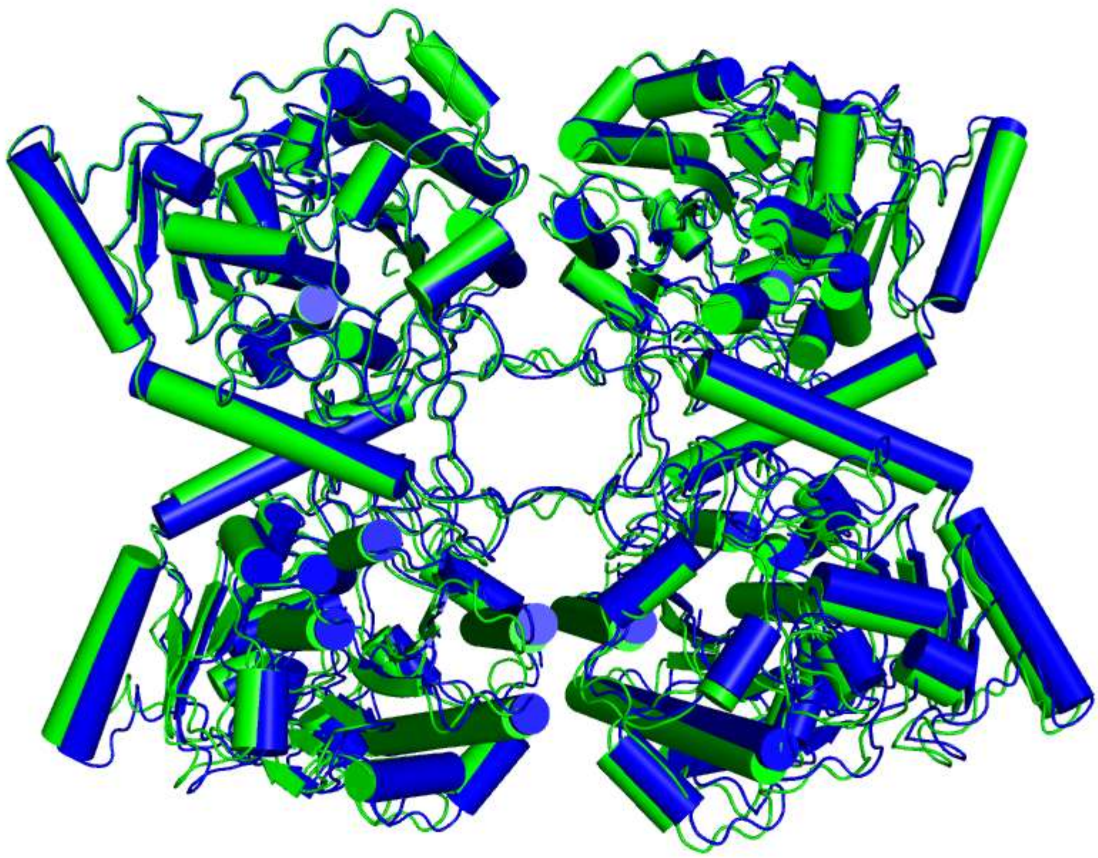

Figure S7. Superposition of the tetrameric structures obtained for the choline-bound WT (PDB 6G60, SG *C*121, blue) and C54S SmeCOSe (PDB 7PTH, SG *P*12_11, green). Only a slight rotation of two of the protomers was found, although superposition of the isolated monomers showed no appreciable differences in any of the loops related to the ligand binding environment.

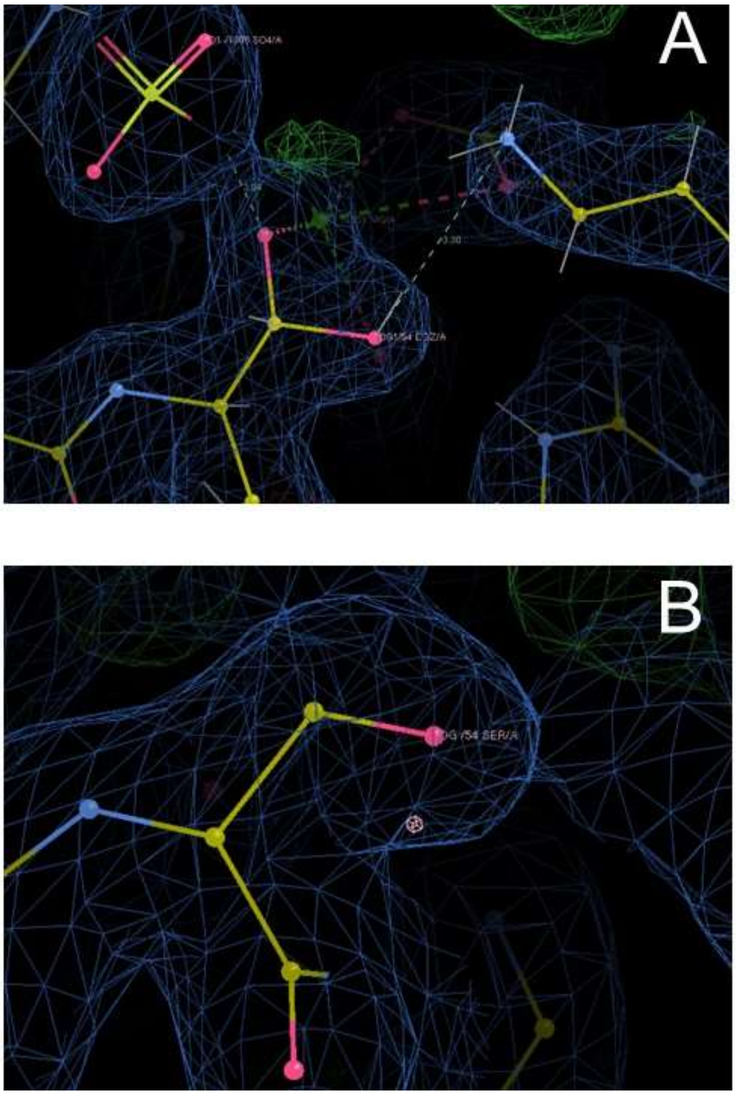

Figure S8. A) Density maps for hydrated formyl-glycine residue and sulfate molecule fitted in the catalytic center of WT SmeCOSe (chain A, PDB 6G5Z). B) Density maps for Ser54 in the C54S SmeCOSe mutant (Chain A, PDB 7PTH). Maps corresponds to the |2Fo-Fc| electron density contoured at 1σ.

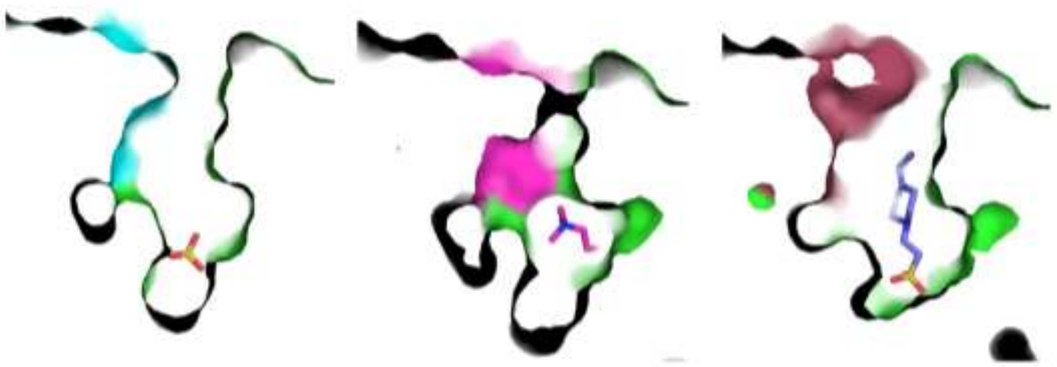

Figure S9. Disposition of sulfate (A), choline (B) and HEPES (C) into the L-shaped tunnel conforming the catalytic cleft of SmeCOSe. The entrance of the tunnel is in the upper part of the figure.

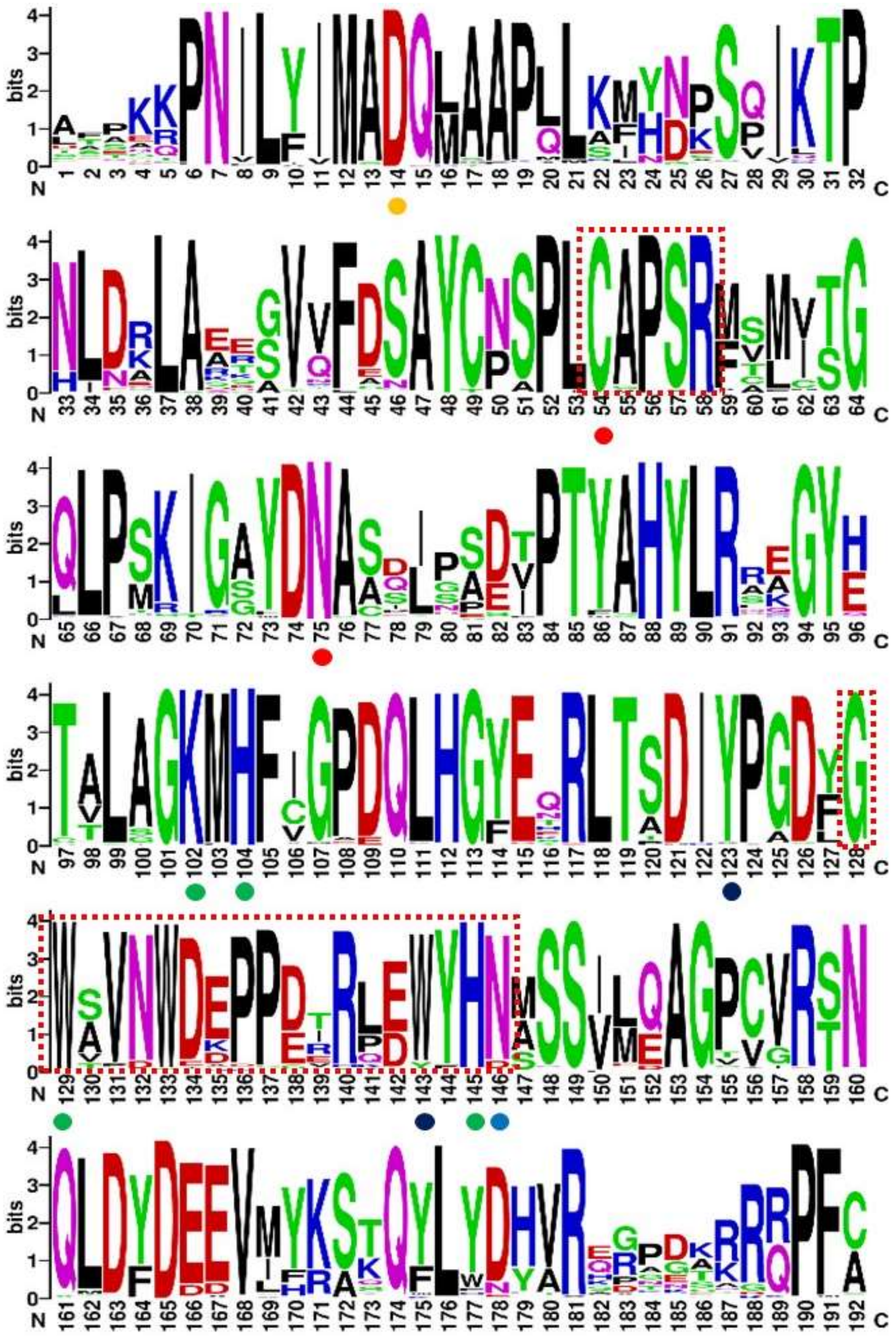

Figure S10. Consensus sequence of several COSe from different origins. A BLAST search was conducted with the SmeCOSe amino acid sequence, excluding *Sinorhizobium* taxa from this search. 500 sequences were retrieved, and they were aligned using ClustalW (Madeira *et al.*, 2019). Phylogenetic analysis was carried out with BioEdit (Hall, 1999). Sequences from the branch containing SmeCOSe (201 sequences) were retrieved, and aligned again with BioEdit. Weblogo was used to plot the results (Crooks et al., 2004); numbering shown in the images is that for SmeCOSe. The position of residues discussed in the main text are marked with a circle.

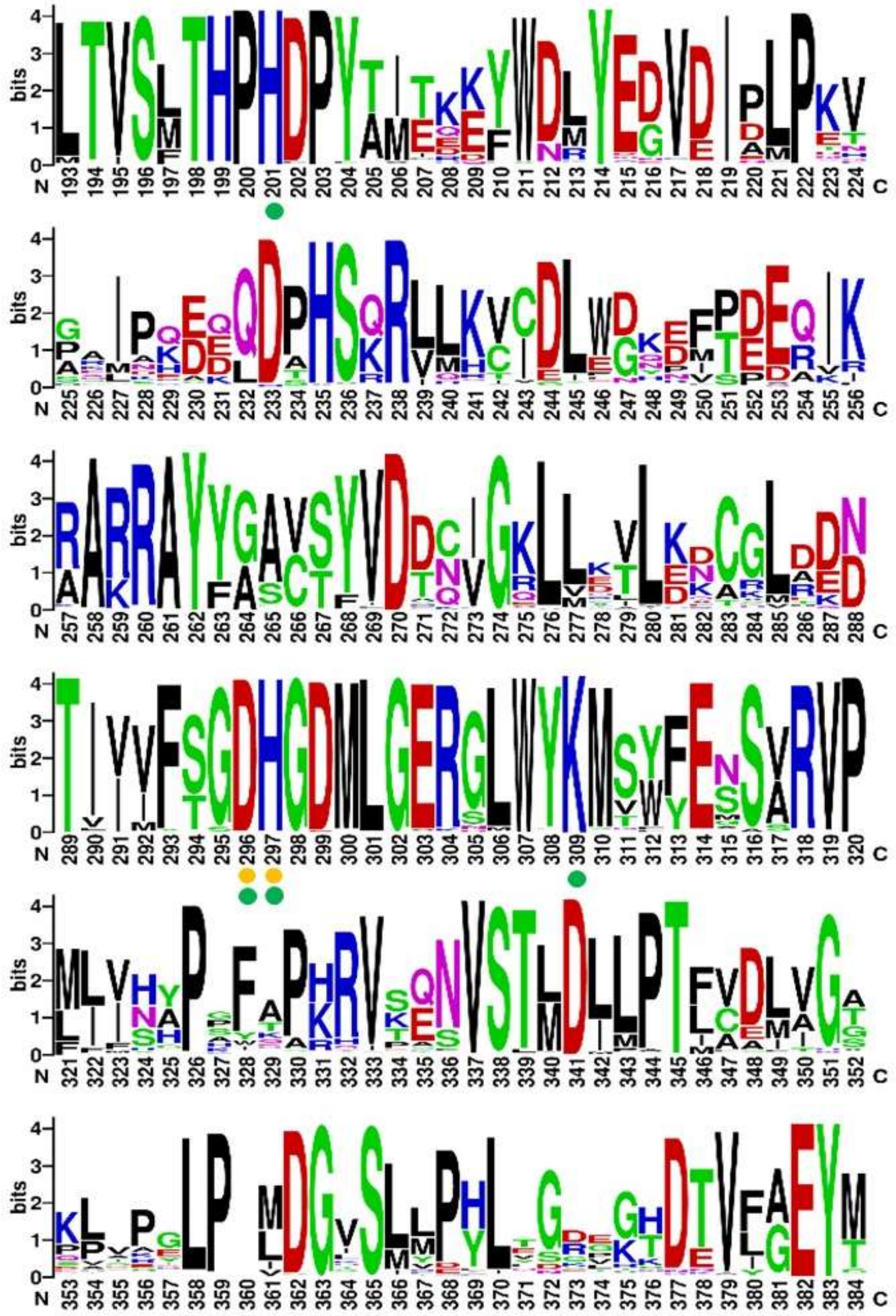

Figure S10. Consensus sequence of several COSe from different origins. (Continuation)

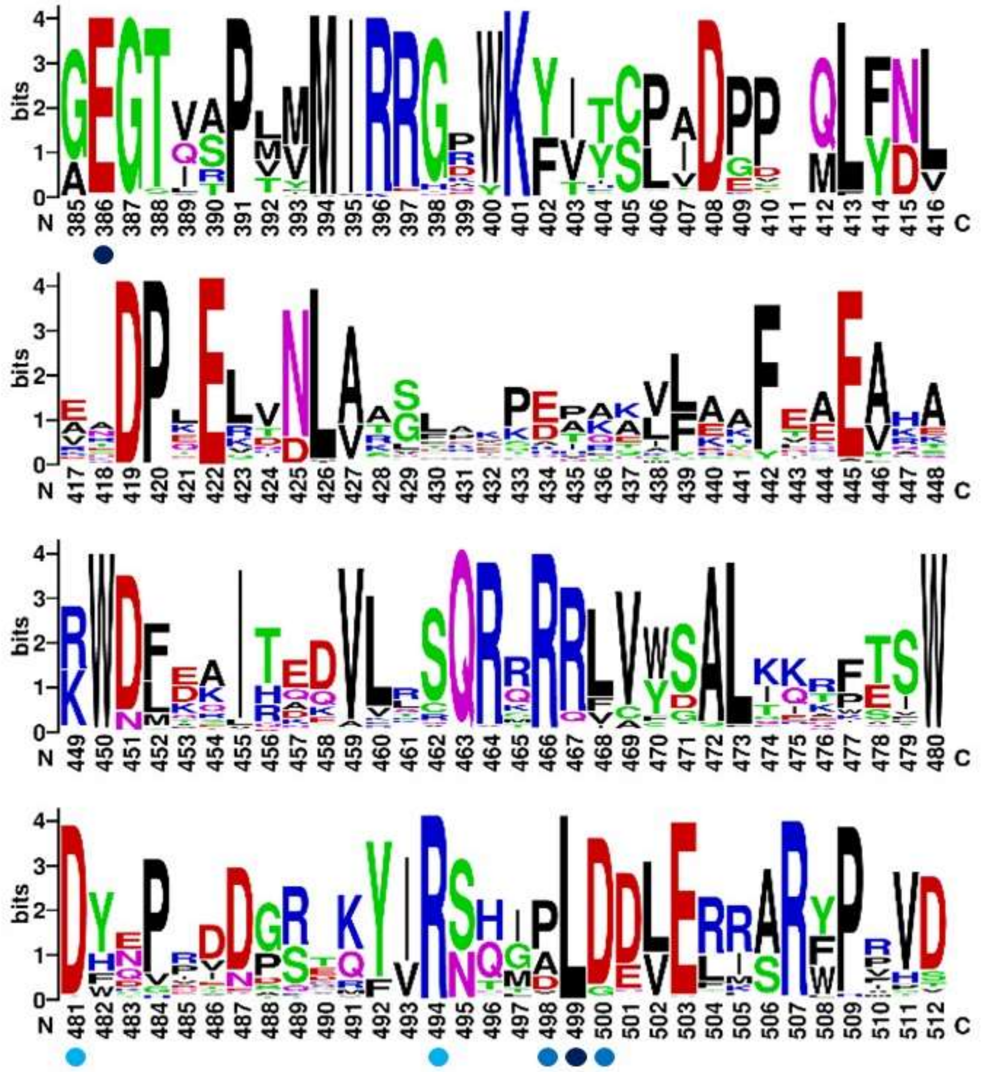

Figure S10. Consensus sequence of several COSe from different origins.
(Continuation)

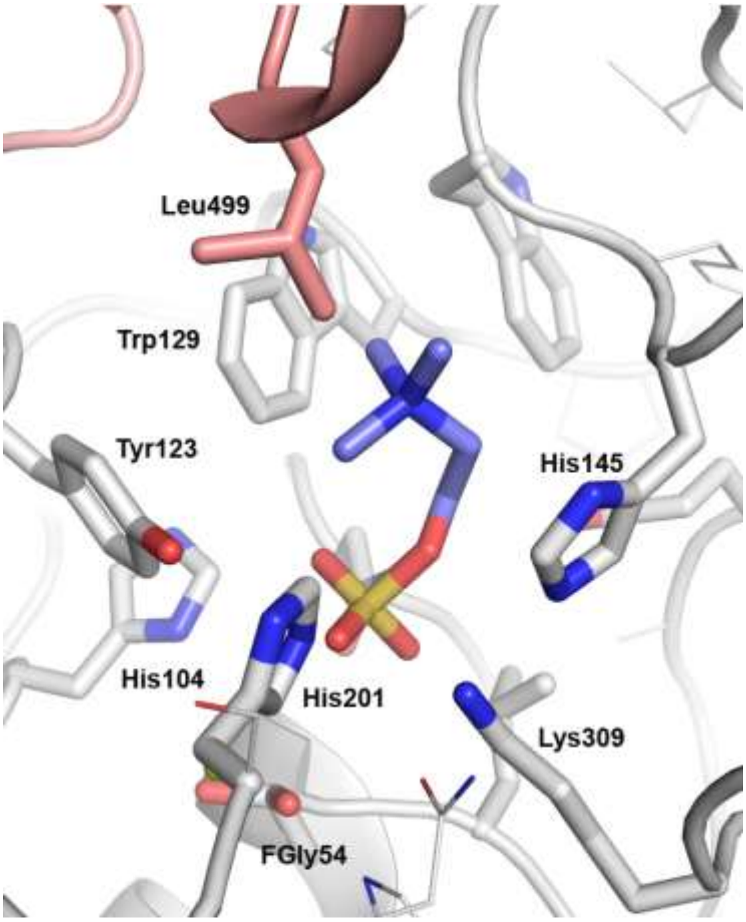

Figure S11. Model generated for COS binding to SmeCOSe based on our different structures. Residues at less than 3.5Å of the modelled COS are Asn75, His201, Lys309 and Leu499 (in red, coming from and adjacent monomer). His104 is also at 3.5 Å of the sulfate moiety of COS in the modelled structure.

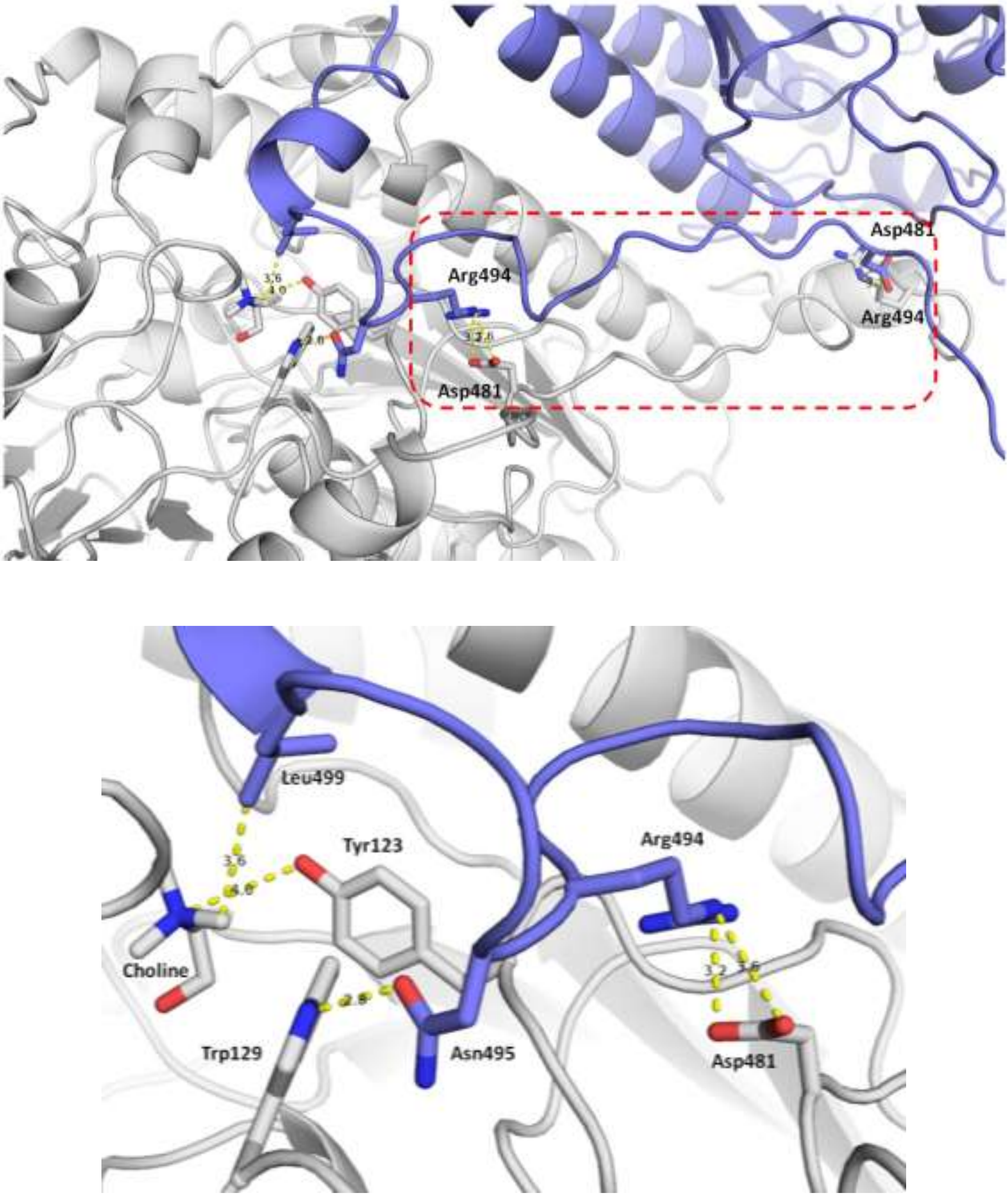

Figure S12. Interactions found among the different subunits of SmeCOSe through the C-terminal extensions. Asp481 and Arg494 most likely “fasten” both ends of this segment. The interaction Trp129-Asn495 connects both sections.

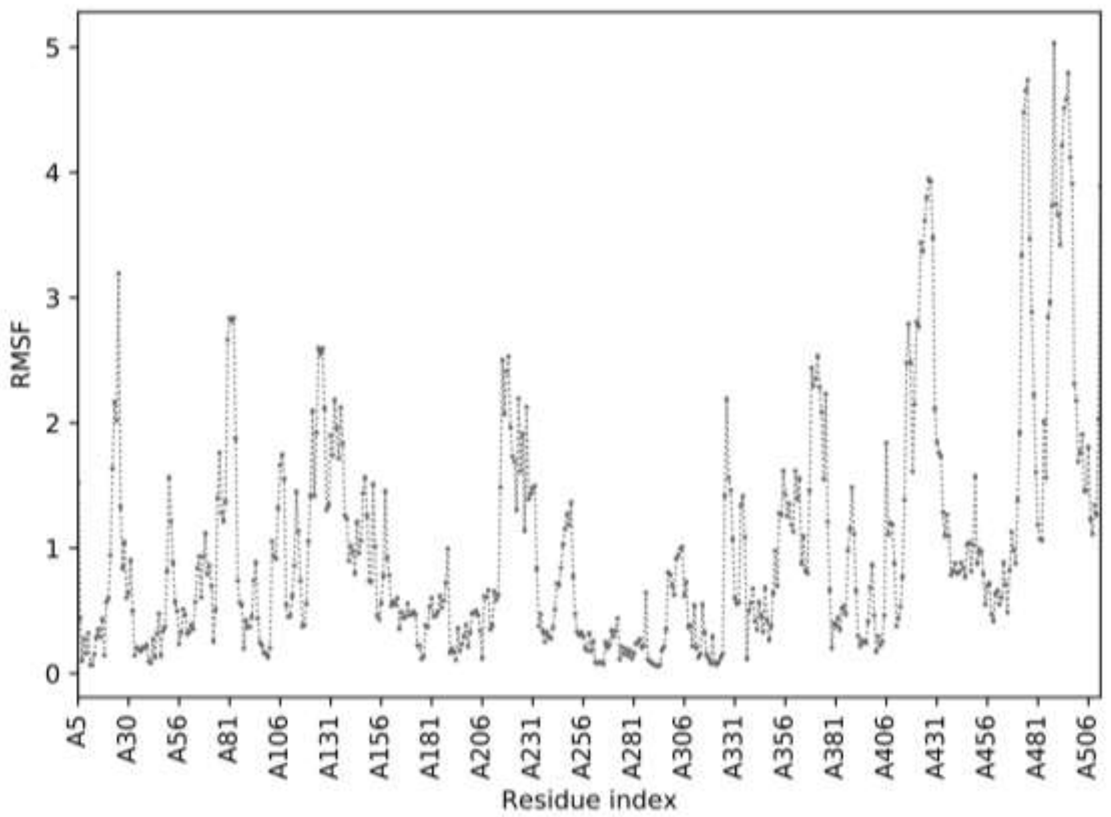

Figure S13. Fluctuation map obtained with the CABS-flex 2.0 server using the sulfate-bound WT SmeCOSe structure as input.

PDB	Z	RMSD	lali	nres	%id	PDB description
4UG4	64.8	0.4	508	515	100	Choline sulfatase
2W8S	37.7	2.5	401	513	27	Phosphonate monoester hydrolase
6PT4	37.4	2.5	411	472	25	Exo-2s-iota carrageenan s1 sulfatase
4UPK	37.2	2.7	400	506	26	Phosphonate monoester hydrolase
2VQR	36.9	2.5	401	512	29	Putative sulfatase
2QZU	36.9	2.4	376	465	26	Putative sulfatase yidj
6S21	36.6	2.4	376	486	22	Endo-4-o-sulfatase
4UPH	36.5	2.7	401	504	27	Sulfatase (sulfuric ester hydrolase) protein
6HHM	36.3	2.8	401	459	23	Arylsulfatase
5FQL	36.2	2.5	383	507	26	Iduronate-2-sulfatase
4UPL	36.1	2.6	401	555	26	Sulfatase family protein
4UPI	36.1	2.4	404	548	26	Sulfatase family protein
6J66	35.9	2.6	377	476	26	Chondroitin sulfate/dermatan sulfate 4-o-endosulfatase
6HR5	35.2	2.0	341	376	25	Alpha-l-rhamnosidase/sulfatase (GH78)
5G2V	34.9	2.5	397	494	23	N-acetylglucosamine-6-sulfatase
3B5Q	34.8	2.2	389	467	26	Putative sulfatase yidj
6UST	34.2	2.6	374	464	26	N-acetylgalactosamine 6-sulfate sulfatase
6PSM	34.0	2.5	352	447	26	Exo-4s-kappa carrageenan s1 sulfatase
3ED4	33.7	2.8	367	474	28	Arylsulfatase
6B0J	33.7	3.0	366	453	26	Iota-carrageenan sulfatase
6USS	33.6	2.6	363	485	23	Sulfatase
1P49	32.3	3.0	367	549	27	Steryl-sulfatase
1FSU	32.2	2.9	370	475	22	N-acetylgalactosamine-4-sulfatase
4FDI	32.1	2.9	364	494	24	N-acetylgalactosamine-6-sulfatase
1N2L	32.0	3.1	368	483	26	Arylsulfatase a
4MIV	31.7	2.5	364	484	24	N-sulphoglucosamine sulphohydrolase
7AJ0	30.8	3.2	354	501	23	Arylsulfatase
4CYS	30.0	3.0	370	534	27	Arylsulfatase
6S20	30.0	2.8	349	480	24	N-acetylgalactosamine-6-o-sulfatase
6XLP	29.7	2.6	311	586	20	LPS-binding protein
6V8Q	28.2	2.7	302	569	18	Inner membrane protein yejm
4UOP	27.1	2.9	322	409	19	Lipoteichoic acid primase
2GSN	26.9	2.5	250	382	20	Phosphodiesterase-nucleotide pyrophosphatase
4TN0	26.0	2.8	268	308	14	UPF0141 protein yjdb
4GTW	25.5	2.8	264	706	16	Ectonucleotide pyrophosphatase/phosphodiesterase
4B56	25.5	2.8	263	816	17	Ectonucleotide pyrophosphatase/phosphodiesterase
6XKD	25.4	2.8	264	681	16	Ectonucleotide pyrophosphatase/phosphodiesterase
6F2T	25.4	2.6	262	722	15	Ectonucleotide pyrophosphatase/phosphodiesterase
4UOR	25.3	2.7	319	418	18	Lipoteichoic acid synthase
2W5Q	25.1	2.9	322	424	17	Processed glycerol phosphate lipoteichoic acid
5YLE	25.0	3.0	270	331	17	Probable phosphatidylethanolamine transferase mcr
4LQY	25.0	2.6	253	379	13	Bis(5'-adenosyl)-triphosphatase enpp4
5MX9	25.0	2.9	267	324	16	Phosphatidylethanolamine transferase mcr-2
5ZZU	24.9	3.1	298	357	15	Phosphoethanolamine transferase eptc
6BNE	24.9	2.8	265	336	15	Phosphoethanolamine transferase

2W8D	24.6	2.8	319	421	16	Processed glycerol phosphate lipoteichoic acid sy
5EGH	24.5	2.6	254	393	16	Ectonucleotide pyrophosphatase/phosphodiesterase
5FGN	24.5	3.1	270	536	13	Lipooligosaccharide phosphoethanolamine transferase
3LXQ	24.4	3.3	327	409	19	Uncharacterized protein vp1736
5TJ3	24.1	2.6	264	520	16	Alkaline phosphatase PAFA
5OLB	21.9	2.6	250	785	16	Ectonucleotide pyrophosphatase/phosphodiesterase
4N7T	21.1	2.7	226	402	18	Phosphopentomutase
4NWJ	20.5	3.0	232	504	17	2,3-Bisphosphoglycerate-independent phosphoglycerate mutase
2IFY	20.5	3.0	229	508	14	2,3-Bisphosphoglycerate-independent phosphoglycerate mutase
3M8W	20.5	2.9	226	391	18	Phosphopentomutase
1O98	20.4	3.0	233	509	15	2,3-Bisphosphoglycerate-independent phosphoglycerate mutase
5KGN	20.1	3.2	232	530	17	2,3-Bisphosphoglycerate-independent phosphoglycerate mutase

Table S1. Dali search (Holm, 2020) using SmeCOSe structure (PDB 6G6Z, chain A). Matches against PDB90 are shown, and with a Z value threshold of 20.

References

Crooks, G.E., Hon, G., Chandonia, J.M. & Brenner S.E. (2004) *Genome Research* **14**, 1188-1190,

Hall, T. A. (1999). *Nucleic Acids Symposium Series* **41**, 95-98.

Holm, L. (2020) *Prot Sci* **29**: 128-140.

Madeira, F., Park, Y. M., Lee, J., Buso, N., Gur, T., Madhusoodanan, N., Basutkar, P., Tivey, A. R. N., Potter, S. C., Finn, R. D. & Lopez R. (2019). *Nucleic Acids Res* **47**(W1), W636-W641.
